# Species integrity, introgression, and genetic variation across a coral reef fish hybrid zone

**DOI:** 10.1002/ece3.6769

**Published:** 2020-10-21

**Authors:** Ashton Gainsford, Geoffrey P. Jones, Jean‐Paul A. Hobbs, Franz Maximilian Heindler, Lynne van Herwerden

**Affiliations:** ^1^ ARC Centre of Excellence for Coral Reef Studies James Cook University Townsville QLD Australia; ^2^ Faculty of Science and Engineering James Cook University Townsville Australia Australia; ^3^ School of Biological Sciences The University of Queensland Brisbane QLD Australia; ^4^ KU Leuven Leuven Belgium

**Keywords:** *Amphiprion leucokranos*, anemonefish, ecological genetics, hybridization, size‐based hierarchy

## Abstract

Hybridization and introgression are evolutionarily significant phenomena breaking down species boundaries. “Hybrid zones” (regions of species overlap and hybridization) enable quantification of hybridization frequency and examination of mechanisms driving and maintaining gene flow. The hybrid anemonefish *Amphiprion leucokranos* is found where parent species (*A. chrysopterus; A. sandaracinos*) distributions overlap. Here, we examine geographic variation in hybridization and introgression, and potential impacts on parent species integrity through assessing relative abundance, social group composition, and genetic structure (mtDNA cytochrome *b*, 21 microsatellite loci) of taxa at three hybrid zone locations: Kimbe Bay (KB) and Kavieng (KA), Papua New Guinea; the Solomon Islands (SO). Relative abundances of and size disparities between parent species apparently drive hybridization frequency, introgression patterns, and genetic composition of taxa. Conspecific groups are most common in KB (65%) where parent species are similarly abundant. Conversely, mixed species groups dominate SO (82%), where *A. chrysopterus* is more abundant. Hybrids most commonly cohabit with *A. sandaracinos* in KB (17%), but with *A. chrysopterus* in KA (22%) and SO (50%). Genetic differentiation (nDNA) analyses indicate that parent species remain distinct, despite ongoing hybridization and hybrids are genetically similar to *A. sandaracinos—*resulting from persistent backcrossing with this smallest species. This study shows that hybridization outcomes may depend on the social and ecological context in which taxa hybridize, where relative abundance and disparate size of parent species explain the frequency and patterns of hybridization and introgression in the *A. leucokranos* hybrid zone, reflecting size‐based dominance behaviors of anemonefish social groups.

## Graphical abstract

Geographic variation in hybridization and introgression, and potential impacts on species integrity were examined using relative abundance, social group composition, and genetic structure of three anemonefish taxa at three hybrid zone locations: Kimbe Bay (KB) and Kavieng (KA) in Papua New Guinea; the Solomon Islands (SO). Relative abundances of and size disparities between parent species apparently drive hybridization frequency, introgression patterns, and genetic composition of taxa. Genetic differentiation (nDNA) analyses indicate that parent species remain distinct, despite ongoing hybridization and hybrids are genetically similar to *A. sandaracinos*—resulting from persistent backcrossing with this smallest species; reflecting the size‐based dominance behaviors of anemonefish social groups.

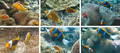

## 1 INTRODUCTION

Hybridization among closely related species is common and can play a significant role in evolution and speciation (Mallet, 2005). Originally thought to be an evolutionary dead end (Coyne & Orr, 2004; Dowling & Secor, 1997; Mayr, 1942), it is now clear that hybridization can contribute to evolutionary change in various ways, most notably through introgression where genetic information from one species transfers to another through repeated backcrossing (Abbott et al., 2013). Hybridization can promote evolutionary novelty within a system faster than through mutation alone (Grant & Grant, 1994; Kunte et al., 2011). The outcomes of hybridization events are diverse, include fusion of species, reinforcement of reproductive barriers, and generation of new distinct populations of mixed ancestry, and may provide the foundation for speciation and diversification to occur (Abbott, Hegarty, Hiscock, & Brennan, 2010; Mallet, 2007,; Meier et al., 2017; Servedio & Noor, 2003; Taylor et al., 2006; Via, 2009; Wu, 2001). Studying young hybrid taxa therefore allows contemporary insights into potential speciation events or species coalescence in progress, occurring at secondary contact zones between closely related taxa that may be undergoing rapid adaptive radiations (Gourbiere & Mallet, 2010; Meier et al., 2017; Price & Bouvier, 2002; Seehausen, 2004). However, in nature the mechanisms driving and maintaining hybridization, determining patterns of introgression, and maintaining species integrity remain poorly understood.

Hybrid zones provide natural laboratories for studying hybridization and investigating patterns of variation among hybridizing species. Hybrid zones may vary spatially and temporally, with taxa subjected to demographic processes in which novel ecological opportunities may arise (Abbott et al., 2013). Ecological factors often associated with hybridization include abundance disparities between closely related taxa and the shared use of a limited resource (i.e., host, food source, and habitat). As such, the causes and consequences of hybrid zones are complex and varied, and patterns of gene flow represent single observations in time of a dynamic interaction between species (Abbott et al., 2013).

Hybridization was once considered rare in the marine environment (Arnold, 1997); however, a surge of recent studies has challenged these traditional perceptions of hybrid scarcity (Gardner, 1997; Harrison et al., 2017; He, Johansen, Hoey, Pappas, & Berumen, 2019; Johansen et al., 2017; Montanari, Hobbs, Pratchett, Bay, & van Herwerden, 2017; Montanari, Hobbs, Pratchett, & van Herwerden, 2016; Pazmiño et al., 2019; Willis, van Oppen, Miller, Vollmer, & Ayre, 2006). Hybridization is particularly common in coral reef fishes, where largely allopatric sister species hybridize at their biogeographic borders. Hybridization appears concentrated at two recognized hybrid hot spots or “suture zones” (Hewitt, 2000; Remington, 1968). The biogeographic border between the Indian and Pacific Oceans, near Christmas and Cocos (Keeling) Islands, marks one region where regionally distinct sister taxa come into contact and hybridize frequently (Hobbs & Allen, 2014; Hobbs, Frisch, Allen, & van Herwerden, 2009; Marie, van Herwerden, Choat, & Hobbs, 2007). At the Christmas Island hybrid hot spot, fifteen hybrid fishes involving 27 species across eight families have been confirmed (Hobbs & Allen, 2014). The other recognized marine suture zone, the Socotra Archipelago, where fourteen putative hybrid coral reef fishes from four families have been recorded (DiBattista et al., 2015), is the junction of four marine biogeographic provinces (Red Sea—Gulf of Aden, Arabian Sea, western Indian Ocean and greater Indo‐Polynesian). Suture zones where hybridization occurs provide ideal environments to address evolutionarily important questions.

The line of convergence between Indo‐Australian and Pacific plates from north‐western Papua New Guinea (PNG) to the Solomon Islands (SO) represents a third, but lesser known suture zone ("PNG‐Solomon Islands suture zone") where the ranges of many sister species also overlap and taxa hybridize (Gainsford, van Herwerden, & Jones, 2015; Hobbs, van Herwerden, Pratchett, & Allen, 2013; McMillan, Weigt, & Palumbi, 1999). For example, the two butterflyfish species *Chaetodon punctatofasciatus* and *C. pelewensis* commonly hybridize here, where McMillan et al. (1999) found a greater frequency of hybrid phenotypes in comparison with parental phenotypes, suggesting greater fitness of hybrids to parental species within the hybrid zone. The "PNG‐Solomon Islands suture zone" has a dynamic history of disturbance associated with climatic changes and sea level fluctuations. It falls within the eastern part of the Coral Triangle—the global center of marine biodiversity (Hughes, Bellwood, & Connolly, 2002)—where many closely related species share habitats, increasing hybridization opportunities. Thus, the ‘PNG‐Solomon Islands suture zone’ can provide unique insights into processes promoting hybridization between cohabiting species and how hybridization affects biodiversity on coral reefs.

Anemonefishes are an evolutionarily young, rapidly diversifying group that is prone to hybridization (Santini & Polacco, 2006; Timm, Figiel, & Kochzius, 2008), providing an ideal system to test evolutionary questions on hybridization (Abbott et al., 2013). Anemonefish groups are structured based on size, where individuals queue to breed (Buston, 2004; Buston & Cant, 2006). Females are largest and dominant, followed in size by subdominant males, and progressively smaller nonbreeding subordinates (Fricke, 1979; Hattori, 1991). In the ‘PNG‐Solomon Islands suture zone’ hybridization occurs between anemonefish species *Amphiprion chrysopterus* and *Amphiprion sandaracinos* which cohabit the same anemone host species (Gainsford et al., 2015). Due to distinctive colouration, the hybrid of these species was initially described as a nominal species, *A. leucokranos*, but later confirmed to be a hybrid based on intermediate morphology along with genetic and ecological similarities (Gainsford et al., 2015). The parent species have predominantly allopatric distributions but cohabit and hybridize within a narrow area of overlap, hereafter termed the *A. leucokranos* hybrid zone. Size differences between hybridizing species in the context of anemonefish hierarchical behavior and protandrous hermaphroditism were most important in driving ecological and evolutionary patterns observed in this hybridization. The larger *A. chrysopterus* always mates as the female when reproducing with the smaller *A. sandaracinos* (Gainsford et al., 2015). Further, the intermediate sized hybrid was always female when backcrossing with the significantly smaller parent species, *A. sandaracinos*. This raises the question as to what drives the frequency of hybridization and backcrossing in the *A. leucokranos* hybrid zone and what maintains species integrity in this dynamic region.

Factors such as abundance disparities, overlapping patterns of resource use, and breakdown in assortative mating all promote hybridization in marine fishes (Montanari et al., 2016); however, these factors may vary across the hybrid zone. To understand which underlying mechanisms maintain the hybrid zone and integrity of hybridizing species, knowledge of the geographic abundance patterns of parent species; levels of cohabitation of parent species and hybrids within anemone hosts, and patterns of backcrossing and introgression are required. If relative abundances of hybridizing species differ or cohabitation levels change, the prevalence of hybrids and introgression levels between species is likely to differ strikingly. Furthermore, geographic variation in genetic differentiation levels between parent species may impact on interbreeding propensity and/or the magnitude of introgression between species.

The overall aim of this study was to investigate geographic variation in abundance, cohabitation, phenotypic characteristics, and genetic composition of parent species and hybrids across the *A. leucokranos* hybrid zone. A combination of ecological observations, phenotypic measurements, and genetic analyses (using mitochondrial and nuclear DNA markers) was applied to answer five specific questions: (1) How does relative abundance of parent species, hybrid frequency, and cohabitation among taxa vary across the hybrid zone? (2) Are hybrid phenotypic characteristics and likely patterns of backcrossing based on phenotypic characters consistent across the hybrid zone? (3) Does host anemone use by these taxa vary across the hybrid zone? (4) Are patterns of historic (mtDNA) and contemporary (nDNA) genetic structure among parent species and hybrids consistent across the hybrid zone or do patterns differ? and (5) How does genetic structure of taxa across the hybrid zone relate to parent species regional abundances? Answering these questions will inform of mechanisms promoting and maintaining hybridization, pathways of introgression, and likely consequences thereof to the resilience of hybridizing taxa into the future.

## 2 MATERIALS AND METHODS

### 2.1 Study taxa and locations

**Figure 1:**
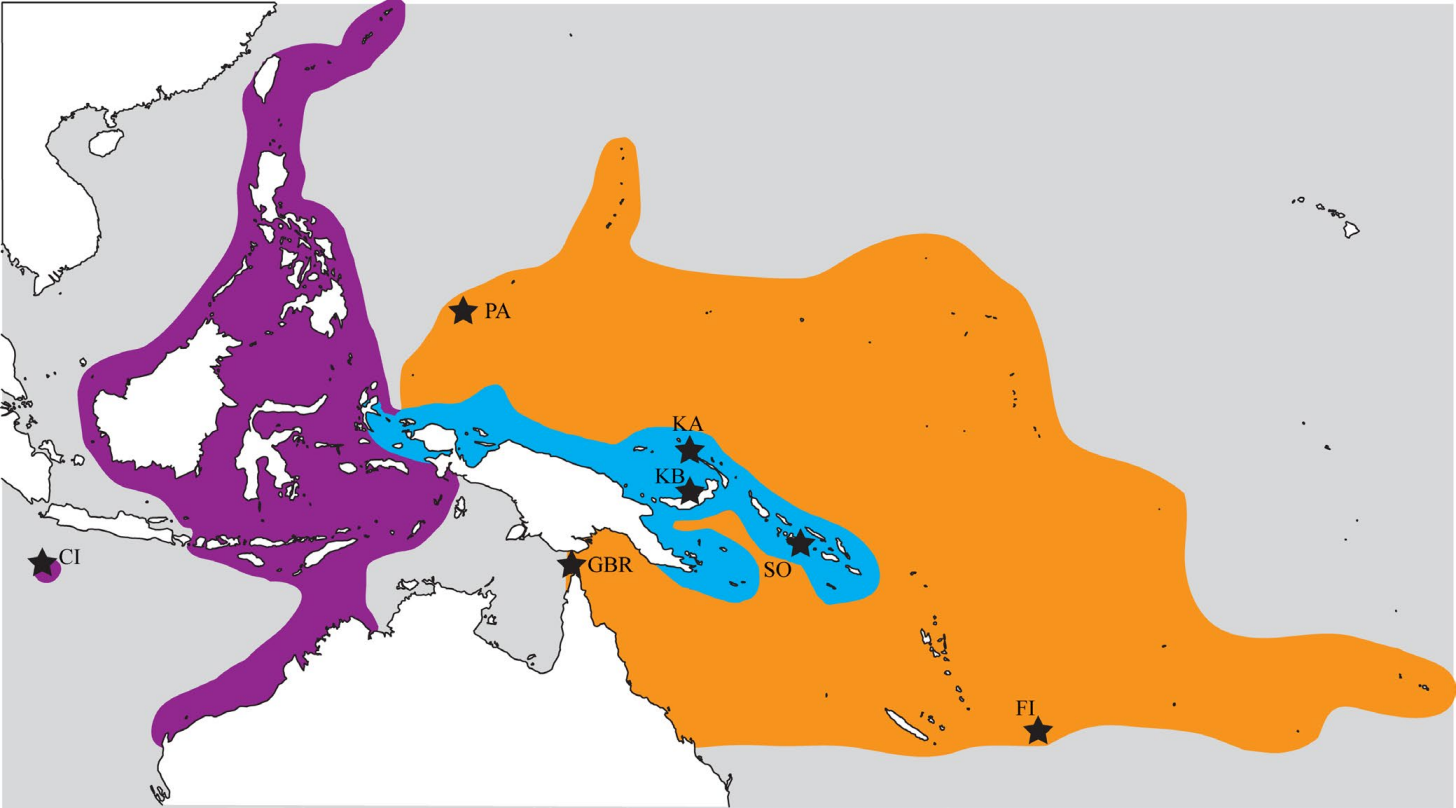
Distribution map indicating sampling sites (black stars) within the *Amphiprion leucokranos* hybrid zone (blue), where species known biogeographical distributions overlap: *A. sandaracinos* (purple), *A. chrysopterus* (orange), and *A. leucokranos* (hybrid, blue). Sites abbreviated as follows: Christmas Island (CI), Palau (PA), Great Barrier Reef (GBR), Kimbe Bay (KB), Kavieng (KA), Solomon Island (SO), and Fiji (FI)

**Figure 2:**
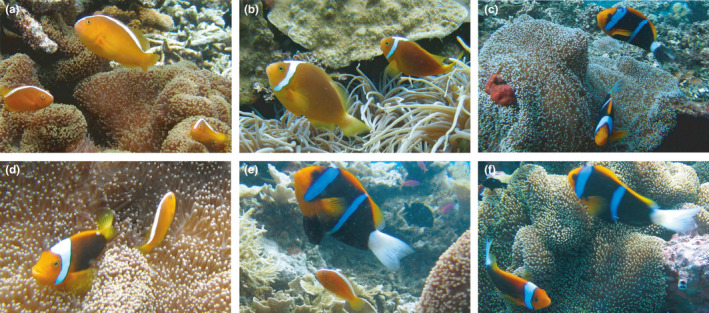
Study species group combinations found within the hybrid zone including: (a), "pure" *A. sandaracinos*; (b) hybrid *A. leucokranos* only (observed with egg clutch); (c) "pure" *A. chrysopterus* (egg clutch indicated with white arrow; note pigmentation of top individual), (d) hybrid *A. leucokranos* with *A. sandaracinos*, (e) *A. chrysopterus* with *A. sandaracinos*, and (f) a putative *A. chrysopterus* and "*A. leucokranos*" hybrid with *A. chrysopterus*. Note host anemone species are *Stichodactyla mertensii* (a, c‐f) and *Heteractis crispa* (b). Photo credits: A. Gainsford

This study was conducted at sites within the *A. leucokranos* zone between 2011 and 2014 (Figure 1). The yellow anemonefish, *Amphiprion sandaracinos* (Figure 2a), occurs from Japan south to the Solomon Islands and west to north‐western Australia and Christmas Island (Indian Ocean). The orange‐fin anemonefish, *Amphiprion chrysopterus* (Figure 2c), occurs throughout the Pacific from Palau, Papua New Guinea (PNG) and northern Great Barrier Reef in Australia, eastward to French Polynesia (Fautin and Allen, 1997). The *A. leucokranos* hybrid zone is found where these parent species distributions overlap, along the northern PNG coastline to the Solomon Islands (104′N, 12842′E to 1050′S, 16228′E). Within the hybrid zone, the two parent species form heterospecific groups and various novel hybrid phenotypes are present. In Kimbe Bay (PNG), hybrid phenotypes range from directly intermediate to parent species phenotypes (Figure 2b) to phenotypes often resembling *A. sandaracinos*, and rarely *A. chrysopterus* (Gainsford et al., 2015).

Abundance, cohabitation patterns, phenotypic characteristics, and genetic composition were examined at three locations within the hybrid zone: (1) Kimbe Bay—KB (530′S, 15005′E), New Britain, PNG; (2) Kavieng—KA (236′S, 15,041′E), New Ireland, PNG; and (3) southern New Georgia islands (845′S, 15815′E), Solomon Islands—SO (Figure 1). Anemonefish groups were opportunistically sampled due to patchy distribution and relative rarity of anemones, resulting in samples from 43 reef sites across all locations. Additionally, outside the hybrid zone, representative samples from "pure" populations of parent taxa were collected for use in mtDNA phylogenetic analysis. *Amphiprion sandaracinos* were collected from Christmas Island (1030′S, 10540′E) and *A. chrysopterus* from Palau (705′N, 13415′E), Fiji (1829′S, 17808′E), and north eastern Australia (1628′S, 14801′E). Fish were captured using hand nets, anaesthetized in situ using clove oil, and released into their home anemone postsampling.

### 2.2 Abundance and cohabitation

All individuals of the parent species *A. chrysopterus* and *A. sandaracinos*, and hybrid *A. leucokranos* observed at the three locations were recorded. Levels of cohabitation were compared by recording: (1) The number of individuals of conspecific *A. chrysopterus* groups, (2) conspecific *A. sandaracinos* groups, (3) hybrid only groups, (4) heterospecific *A. chrysopterus* and *A. sandaracinos* groups, (5) heterospecific *A. chrysopterus* and hybrid groups, (6) heterospecific *A. sandaracinos* and hybrid groups, and (7) groups containing both parents and hybrids. Additionally, habitat use by parent species and hybrids were characterized among regions within the hybrid zone. Most individuals were encountered between 1 and 20 m depth, where depth, host anemone species, immediate surrounding habitat, and reef zone (reef flat, crest, and slope) were recorded for all groups examined in this depth zone. For each individual captured, the following data were recorded: phenotype (photographed), total length (measured to the nearest mm), and sex (assigned based on relative social position). The presence of egg clutches was recorded when observed, and putative parent species identified.

### 2.3 Phenotypic characteristics

The relative frequency of hybrid phenotypes was calculated from photographs using seven qualitative traits including: tail shape and color, dominant body color, presence and completeness of dorsal stripe and side bars, as well as latitudinal body shape (see Table S1 for phenotypic categories ranging from pure *A. chrysopterus* to hybrids, and pure *A. sandaracinos*, and Table S2 for relative frequency of qualitative phenotypic traits).

### 2.4 Genetic structure

The population genetic and phylogenetic structure within and outside the hybrid zone was compared to assess regional variation in hybridization propensity among regions. While anaesthetized, each individual was fin‐clipped for genetic analyses. Small (4 mm^2^) caudal fin clips were taken from all captured fish and preserved in 80% ethanol. Samples of "pure" parental species from outside the hybrid zone were included to allow for comparison of species‐specific genetic signals. Both mitochondrial cytochrome *b* and nuclear microsatellite markers were employed to estimate historical and contemporary gene flow, respectively.

#### 2.4.1 Laboratory protocols for genetic analyses

For all laboratory methods described herein, genomic DNA was isolated from fin clips using a standard salting out protocol (Sunnucks & Hales, 1996), and polymerase chain reaction (PCR) products were column purified with GE Illustra Sephadex G‐50 for sequencing.

Approximately a 430 bp mitochondrial cytochrome *b* gene fragment was amplified from parent species and hybrids using universal primers (CB3H; 5′‐GGCAAATAGGAARTATCATTC‐3′ and L15162; 5′‐GCAAGCTTCTACCATGAGGACAAATATC‐3′) following amplification procedures in Gainsford et al. (2015), where purified PCR products were sequenced with each primer using ABI technologies (Macrogen, South Korea).

Forty‐two *Amphiprion* spp. microsatellite markers, including 8 novel loci, were tested on seven to eight individuals each of *A. sandaracinos*, *A. chrysopterus* and hybrid taxa. Novel primer development and cross‐amplification success of markers is detailed elsewhere (Gainsford, Jones, Gardner, & van Herwerden, 2020). Of all markers tested, 23 highly polymorphic loci that consistently cross‐amplified in all study taxa across regions were used in optimized multiplex reactions, based on locus sizes using Multiplex Manager 1.0 software (Holleley & Geerts, 2009). PCRs of seven multiplex sets of two to six markers were carried out in 10 µl reactions with 50 ng template, 2X Type‐it Multiplex PCR Master Mix (QIAGEN), and 2 µM each primer (forward and reverse). PCR products were visualized by gel electrophoresis using 2.0% agarose, purified as above, and genotyped on an ABI 3730XL Genetic Analyser (Applied Biosystems) with GeneScan LIZ‐600bp size standard.

#### 2.4.2 Data compilation and analyses

Cytochrome *b* sequences were MUSCLE aligned (Edgar, 2004a, 2004b) and manually edited in Geneious v9.0.4. An alignment including sequences from all regions sampled, including those outside the hybrid zone, was used to estimate phylogenetic evolutionary history of taxa and relationships among haplotypes. The best substitution model for the alignment was the HKY + G model chosen from 21 models using a likelihood approach under default settings in MEGA6 (Tamura, Stecher, Peterson, Filipski, & Kumar, 2013). Phylogenetic relationships were inferred using standard approaches including maximum parsimony (MP) and maximum likelihood methods in MEGA6 (Tamura et al., 2013), and Bayesian inference (BI) using the MrBayes 3.2 plug‐in (Ronquist et al., 2012) through Geneious (Figures S2–S4). For all analyses, the HKY + G substitution model was implemented, and trees were outgroup rooted using individuals from *Amphiprion ocellaris* (DQ343956‐7, KF264293‐4). MP analyses included 10 independent runs using 1,000 bootstrap replicates, with all ten best MP trees recovered having identical length and topology. ML analyses were performed using 1,000 bootstrap replicates under a likelihood approach, and BI analyses were conducted with 1,100,000 iterations and 100,000 tree burn‐in.

All population mtDNA genetic analyses were performed in Arlequin v3.5.1.2 (Excoffier & Lischer, 2010) to estimate levels of gene exchange between and within populations, where all populations outside the hybrid zone were excluded due to sample sizes < 10. Species sequence sets were defined a priori in DnaSP v5.10.1 (Librado & Rozas, 2009). A minimum spanning tree (MST) was constructed manually and edited in Illustrator (Adobe Systems Inc.) to elucidate patterns of haplotype distribution among and within populations. Genetic diversity indices including haplotype diversity (*h)* and nucleotide diversity (π) were calculated for all populations. Spatial heterogeneity for cytochrome *b* was assessed through population pairwise F_ST_ and analysis of molecular variance (AMOVA) following 1,000 permutations, where the proportion of variance among species groups (F_CT_), the proportion of variation among populations within species groups (F_SC_), and the proportion of variation within populations (F_ST_) were estimated using a pairwise difference model.

Microsatellite genotypes were scored and manually edited using GeneMarker (SoftGenetics, USA). Of the 23 markers tested, 21 could be confidently scored. Population genetic analyses of microsatellite markers were based on a total of 124 *A. chrysopterus*, 122 *A. sandaracinos*, and 113 hybrid (*A. leucokranos*) individuals collected from Kavieng (*n* = 26, 28, 25, respectively), Kimbe Bay (*n* = 31, 66, 35, respectively), and the Solomon Islands (*n* = 65, 30, 53, respectively; Table S3). Sample sizes of populations outside the hybrid zone were too small (*n* < 5) and therefore excluded from further nDNA population genetic analysis. Number of alleles (N_A_), private alleles (P_A_), observed (H_O_), and expected (H_E_) heterozygosities were calculated in Genalex v6 (Peakall & Smouse, 2006, 2012), and the average inbreeding coefficient (F_IS_) was estimated in Arlequin (Excoffier & Lischer, 2010). Probabilities of departure from Hardy–Weinberg equilibrium (HWE) and linkage disequilibrium (LD) were calculated in Genepop v4.0 (Rousset, 2008) using Markov chains with dememorisation of 10,000 with 20 batches of 5,000 iterations per batch. The presence of null alleles, large allelic dropout and scoring bias was estimated using Micro‐checker (van Oosterhout, Hutchinson, Wills, & Shipley, 2004). Raw estimates of population structure were calculated locus‐by‐locus and as an average over 21 loci using analysis of molecular variance (AMOVA) with 10,000 permutations, in Arlequin (Excoffier & Lischer, 2010), as well as genotypic diversity (gd) estimates. An excluding null allele correction (ENA) was carried out in FreeNA with 1,000 bootstrap replicates (Chapuis & Estoup, 2007) to estimate species differentiation corrected for null allele frequencies. SMOGD v1.2.5 (Crawford, 2010) was used to calculate estimates of actual differentiation (D_est_), and Structure v2.3.4 (Pritchard, Stephens, & Donnelly, 2000) was used to estimate the number of differentiated genetic populations (K) represented by samples. Structure was run using the admixture ancestry model informed by location, with correlated allele frequencies for each K value for 10 individual repetitions, at 1,000,000 MCMC iterations following a 100,000 burn‐in. Structure Harvester (Earl & von Holdt, 2012) was used to assess the best K following Evanno's method (Evanno, Regnaut, & Goudet, 2005). To visually assess relationships between predefined population clusters, a discriminant analysis of principle components (DAPC) was executed using the adegenet package (Jombart, 2008) in R v2.13.2 (R Development Core Team, 2016). DAPC retained 198 principle components, accounting for 95% of the variability present, and is visually represented in a scatterplot of the first two principle components with 95% genotypic inertia ellipses (IE) for each population shown.

## 3 RESULTS

### 3.1 Relative abundance and patterns of cohabitation

**Figure 3:**
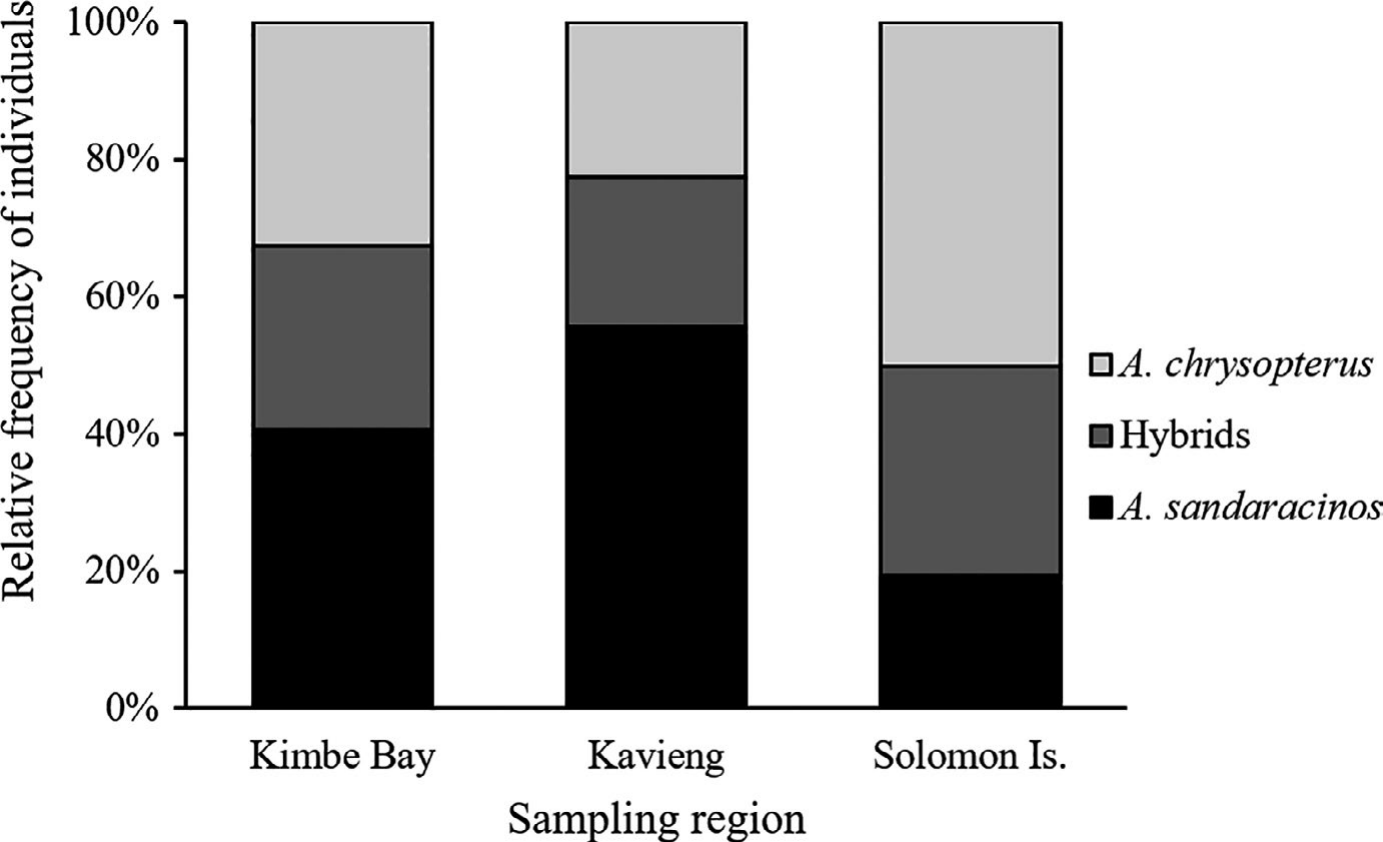
Relative frequency of "pure" *A. chrysopterus*, "pure" *A. sandaracinos* and hybrid (*A. leucokranos*) individuals across the three sampled regions within the hybrid zone—including all ranks from recruits to adults

Across the hybrid zone, the relative frequency of hybrid individuals was comparable among the three surveyed locations (22%–30%; Figure 3). However, when considering parent species, the relative frequency of *A. sandaracinos* was over twofold greater than *A. chrysopterus* in Kavieng (56% and 23%, respectively), in contrast to the Solomon Islands where *A. chrysopterus* was more prevalent than *A. sandaracinos* (50% and 19%, respectively; Figure 3). Comparatively, parent species, *A. chrysopterus* and *A. sandaracinos*, were observed at relatively equal frequency in Kimbe Bay.

**Figure 4:**
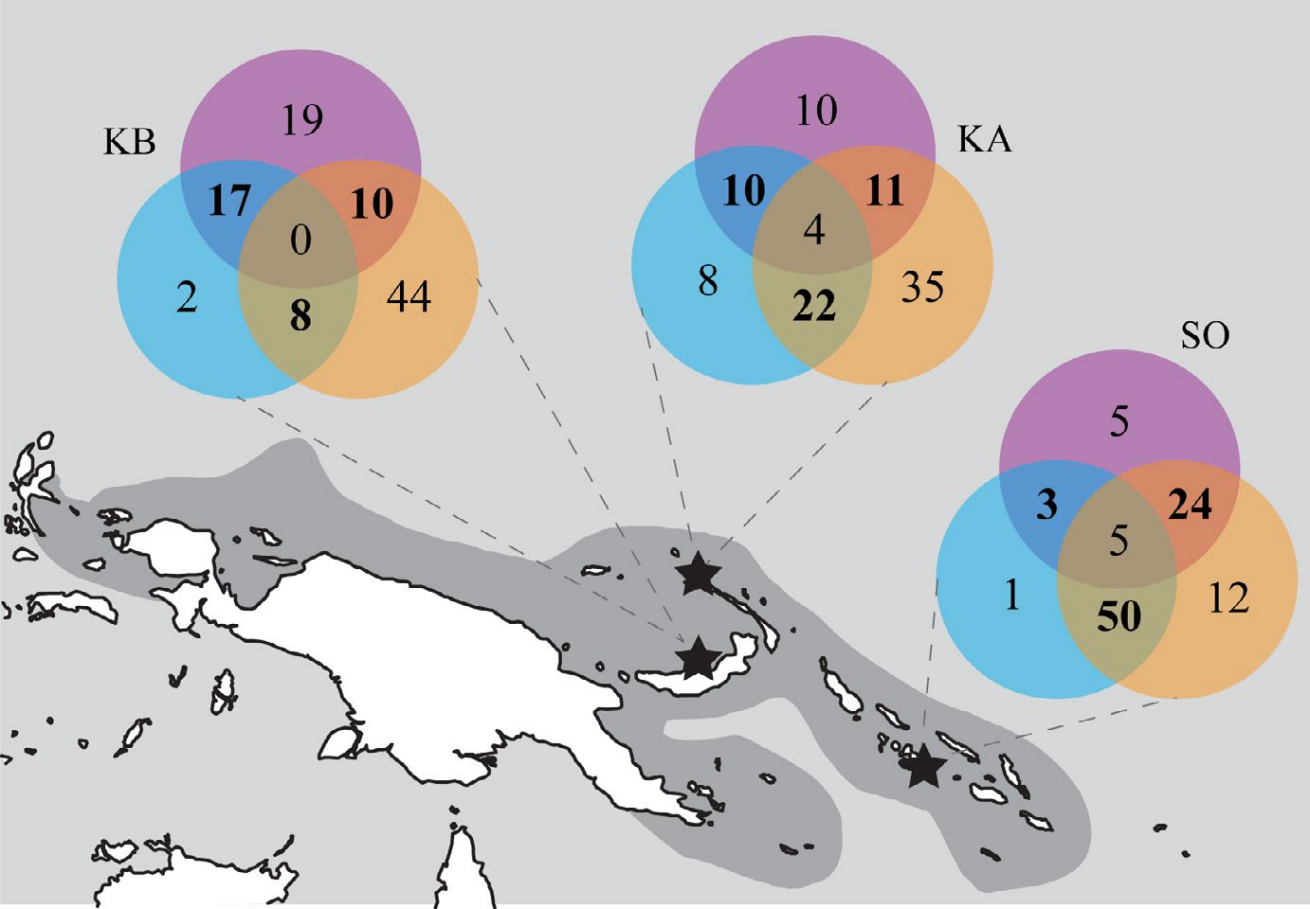
Relative abundance of anemonefish hybrid zone group assemblages (%) for "pure" *A. chrysopterus* (orange), hybrid (blue), and *A. sandaracinos* (purple) groups, as well as mixed taxonomic groups (circle intersects) within the hybrid zone. Total groups sampled: KB (*n* = 101), KA (*n* = 72), and SO (*n* = 77)

All combinations of conspecific and heterospecific groups were observed across all hybrid zone survey locations, including hybrid only groups (Figures 2 and 4). However, the proportion of conspecific versus heterospecific groups varied across the hybrid zone. Conspecific groups were most prevalent in Kimbe Bay (65%), compared with 18% in the Solomon Island region (Figure 4). Thus, 82% of Solomon Islands groups contained heterospecifics. Across all three locations, there was a greater proportion of conspecific *A. chrysopterus* than *A. sandaracinos* groups (12%–44% and 5%–19%, respectively); however, both parent species showed a similar pattern of group composition across the hybrid zone. The proportion of conspecific groups for both *A. chrysopterus* and *A. sandaracinos* was greatest at Kimbe Bay (44% and 19%, respectively) and least at Solomon Islands (12% and 5%, respectively; Figure 4). Therefore, the proportion of heterospecific groups, and thus the incidence of hybridization, varied across the hybrid zone.

The formation of hybrid only groups was low and varied across the hybrid zone: between 8% (Kavieng) and 1% (SO). Interestingly, the proportion of hybrid and *A. chrysopterus* heterospecific groups varied sixfold across the hybrid zone from 8% at Kimbe Bay to 50% at Solomon Islands, whereas the proportion of hybrid and *A. sandaracinos* groups increased at a comparable magnitude in the opposite direction from 3% at Solomon Islands to 17% at Kimbe Bay. Groups containing both parental species and the hybrid were present at Kavieng (4%) and Solomon Islands (5%), but not at Kimbe Bay. This geographic variation in composition of social groups containing hybrids is important to document, because these patterns could lead to differences in the level and direction of introgression across the hybrid zone.

### 3.2 Phenotype variation and relative frequency

Relative frequency of phenotypic traits revealed considerable regional variation in both *A. chrysopterus* and hybrids, but minimal variation across *A. sandaracinos* populations (Table S1). Ninety six percent of Solomon Islands hybrids had an elongated tail shape similar to *A. chrysopterus*, in comparison to 96% of Kimbe Bay and Kavieng hybrid individuals that had an *A. sandaracinos*‐like round tail phenotype (Table S1). Body color was highly variable in *A. chrysopterus*, with black body color most common in Kimbe Bay and Solomon Islands (94% and 79%, respectively), compared with intermediate proportions of black‐ and brown‐colored individuals in Kavieng (52% and 48%, respectively).

The highly variable hybrid phenotypes previously reported at Kimbe Bay (Gainsford et al., 2015) were also found at other examined survey locations in the hybrid zone. During extensive surveys, an additional hybrid phenotype thought to represent a hybrid—*A. chrysopterus* back‐cross was also observed. These individuals had characteristic *A. chrysopterus* body shapes (pectoral and anal fin colouration), in addition to a singular "white bonnet" side bar pattern consistent with *A. leucokranos* hybrid phenotypes (Figure S1d), as well as caudal fin shape and color, blue tinge to white side bar, and facial features consistent with the most common *A. chrysopterus* phenotype in the sampling region. Body colouration faded from dark orange/brown to black. These hybrid—*A. chrysopterus* back‐cross individuals were always present as males paired with *A. chrysopterus* females and displayed particularly bold behavior (*n* = 3). Additionally, a range of *A. chrysopterus* phenotypes were found across the hybrid zone, including brightly pigmented Solomon Island morphs (approximately 9% of population), individuals with half side bars, and notably smaller, light morphs (approximately 2% and 19% of Kimbe Bay and Solomon Island populations, respectively; Figure S1).

### 3.3 Anemone host use

Three species of anemone were used by the anemonefishes studied here, including *Heteractis crispa*, *Stichodactyla mertensii*, and *Heteractis aurora* (rarely). *Amphiprion sandaracinos* almost exclusively inhabited *S. mertensii* (99%, *n* = 226), whereas hybrids (*n* = 181) and *A. chrysopterus* (*n* = 230) consistently used both *S. mertensii* (65% and 61%, respectively) and *H. crispa* (34% and 39%, respectively).

### 3.4 Genetic variation across the hybrid zone

Four hundred and thirty mtDNA cytochrome *b* sites were resolved for 388 individual anemonefishes (Table 1), including 363 individuals from the hybrid zone. Of the 430 cytochrome *b* sites, 184 were polymorphic. Seventy‐two haplotypes were detected in the hybrid zone data set, where 15 haplotypes were shared and 57 were unique, suggesting an accumulation of mutations over time via female mediated gene flow throughout the hybrid zone.

**Table 1:** Marker credentials for Hybrid Zone populations, derived from mtDNA cytochrome *b*: number of individuals (*n*), haplotypes (*n*_h_), haplotype diversity (*h ± SE*), and nucleotide diversity (*π* ± *SE*); and nDNA microsatellites: number of individuals (*n*), alleles per locus (*n*_a_), observed number of private alleles (P_a_), genotypic diversity (gd ± *SE*), observed heterozygosity (H_O_), and expected heterozygosity (H_E_) averaged over 21 loci

Population	mtDNA cytochrome *b*				nDNA microsatellite loci					
	*n*	*n*_h_	h	Π	*n*	*n*_a_	P_a_	gd	H_O_	H_E_
CHKB	76	17	0.819 ± 0.03	0.017 ± 0.01	31	198	8	0.573 ± 0.30	0.556	0.675
CHKA	31	7	0.656 ± 0.06	0.010 ± 0.01	26	179	9	0.624 ± 0.32	0.677	0.715
CHSO	56	9	0.651 ± 0.04	0.010 ± 0.01	65	266	27	0.634 ± 0.33	0.634	0.717
CH overall	163	26	0.739 ± 0.02	0.013 ± 0.01	122					
LUKB	45	13	0.813 ± 0.04	0.028 ± 0.02	35	155	1	0.623 ± 0.32	0.636	0.634
LUKA	23	5	0.640 ± 0.07	0.003 ± 0.00	25	194	3	0.742 ± 0.37	0.765	0.750
LUSO	55	6	0.575 ± 0.03	0.008 ± 0.01	53	258	16	0.735 ± 0.37	0.701	0.757
LU overall	123	18	0.685 ± 0.03	0.015 ± 0.01	113					
SAKB	30	14	0.798 ± 0.07	0.063 ± 0.03	66	160	10	0.478 ± 0.25	0.460	0.503
SAKA	23	12	0.881 ± 0.05	0.073 ± 0.04	28	151	1	0.569 ± 0.29	0.592	0.584
SASO	24	15	0.909 ± 0.05	0.136 ± 0.07	30	122	3	0.507 ± 0.26	0.528	0.556
SA overall	77	37	0.892 ± 0.03	0.102 ± 0.05	124					

Populations of each nominal species (*A. chrysopterus* (CH), *A. leucokranos* (LU) and *A. sandaracinos* (SA)) include Kimbe Bay (KB), Kavieng (KA), and Solomon Islands (SO)

#### 3.4.1 Historical phylogenetic structure (mtDNA)

**Figure 5:**
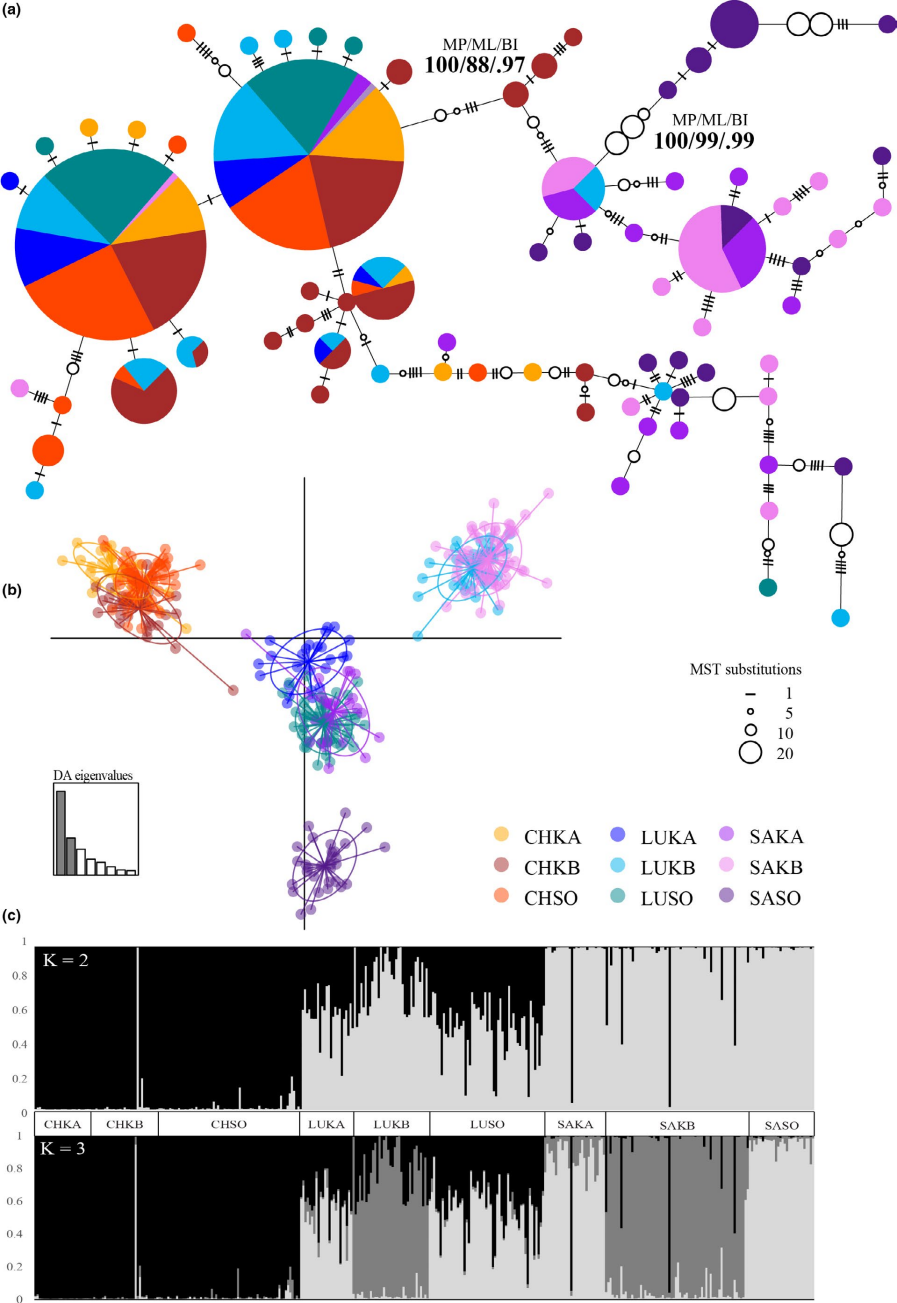
(a) Minimum spanning tree of mtDNA cytochrome b haplotypes from hybrid zone A. sandaracinos, hybrid (*A. leucokranos*) and *A. chrysopterus*, estimated under a median‐joining algorithm. Each "pie" represents an individual haplotype, the size of which is proportional to the total number of individuals sharing that haplotype, where individual population identity is indicated by color according to the key (see inset). Substitutions separating haplotypes are indicated in the legend for one, five, ten, and twenty substitutions, respectively. Phylogenetic relationship structure is inferred from MP and ML bootstrap support values (%), and BI posterior probabilities (HPD, maximum value of 1). See Table 1 for number of individuals per population per species. (b) Scatterplot of DAPC performed on 21 microsatellite loci for 9 populations within the hybrid zone as indicated in the legend. Individual genotypes are represented by dots and population clusters are defined by 95% inertia ellipses. For panels A and B, colors relate to populations with first two letters denoting species, *A. chrysopterus* (CH), *A. leucokranos* (LU), *A. sandaracinos* (SA), and last two letters indicating region, Kavieng (KA), Kimbe Bay (KB), and Solomon Islands (SO). The scree plot (bottom left of panel B) of discriminant analysis (DA) eigenvalues provides a graphical representation of variance of each discriminant function; where shaded bars highlight those retained in analysis. Axes represent the first two discriminant analysis functions. (c) Inferred ancestry of individuals using Bayesian population assignment to K = 2 and K = 3 clusters, as indicated, using 21 microsatellite loci. Each vertical line represents an individual, with proportional genotype assignment to K clusters indicated by different shading

Evolutionary history was inferred using three phylogenetic methods, all producing similar tree topologies with comparable branch lengths (Figures S2–S4). Limited phylogenetic structure was evident, with only a group of Kimbe Bay *A. chrysopterus* (*n* = 5) and Solomon Island *A. sandaracinos* (*n* = 11) delineated from other sequences in all analyses. *Amphiprion chrysopterus* and hybrid populations shared six common haplotypes, with a minority of *A. sandaracinos* representatives, indicating a high level of maternal relatedness of hybrids to the larger parent species, *A. chrysopterus*, throughout the hybrid zone (Figure 5a). *A. sandaracinos* populations share two common haplotypes, one of which is also shared by some Kimbe Bay hybrids providing some evidence for maternal relatedness of *A. sandaracinos* to hybrids in Kimbe Bay only. Rare haplotypes were mostly evident in the Kimbe Bay *A. sandaracinos* population, with *A. sandaracinos* contributing 43% of rare alleles to the hybrid zone overall. Results suggest variation in the degree of mtDNA introgression across regions. Kavieng shows exclusively *A. chrysopterus* female mediated gene flow into *A. sandaracinos* via hybrids. Kimbe Bay shows a similar pattern, reflecting the importance of the size‐based mating hierarchy of anemonefish in mediating gene flow. However, evidence of common *A. sandaracinos* haplotypes shared with limited hybrid individuals in Kimbe Bay suggests extensive backcrossing contributed *A. sandaracinos* mtDNA haplotypes to the hybrid population (Figure 5a). There is no evidence of *A. chrysopterus* mtDNA haplotype introgression into *A. sandaracinos* at the Solomon Islands.

### 3.5 Historical population genetic structure (mtDNA)

The level of population differentiation was high for pairwise population comparisons of cytochrome *b* (Tables 2 and 3), revealing *A. sandaracinos* to be most differentiated from other taxa examined. All "pure" *A. sandaracinos* populations appeared highly differentiated from all other populations, with the exception of Kavieng and Kimbe Bay *A. sandaracinos*, which were not significantly different (Table 2). ‘Pure’ *A. chrysopterus* populations from Solomon Islands and Kimbe Bay were also highly differentiated from each other before and following Bonferroni correction. Similarly, species‐level differences between *A. sandaracinos* compared with *A. chrysopterus* and hybrids were significant (F_ST_ = 0.528 *p* < .0001 and F_ST_ = 0.486 *p* < .0001, respectively), reaffirming historical genetic structure observed in mitochondrial DNA analyses (Figure 5a).

**Table 2:** Pairwise population comparisons for populations within the hybrid zone: F_ST_ (*p*‐value) calculated from 430 bp mitochondrial cytochrome *b* (above diagonal), where significance levels of *p* < .05* before sequential Bonferroni correction, and *p* < .00138 (bold) following the correction are indicated; and the harmonic mean of the estimator of actual differentiation (D_est_) across 21 microsatellite loci (below diagonal)

	CHKB	CHKA	CHSO	LUKB	LUKA	LUSO	SAKB	SAKA	SASO
CHKB		0.008	0.034*	0.006	0.007	0.024*	0.659*	0.578*	0.574*
CHKA	0.006		0.013	0.000	−0.000	−0.004	0.619*	0.530*	0.487*
CHSO	0.013	0.000		0.029*	−0.001	0.010	0.669*	0.597*	0.573*
LUKB	0.591	0.636	0.609		0.011	0.022	0.540*	0.436*	0.452*
LUKA	0.358	0.346	0.33	0.140		−0.017	0.619*	0.534*	0.472*
LUSO	0.292	0.293	0.266	0.176	0.040		0.688*	0.617*	0.580*
SAKB	0.774	0.835	0.809	0.017	0.275	0.309		0.014	0.260*
SAKA	0.788	0.820	0.786	0.107	0.136	0.237	0.082		0.193*
SASO	0.795	0.819	0.791	0.159	0.229	0.194	0.129	0.062	

Sample codes used are as above in Table 1 caption.

**Table 3:** Analysis of molecular variance (AMOVA) for mitochondrial cytochrome *b* and 21 microsatellite loci, respectively, from the three species‐level groups within the hybrid zone

Source of variation	*df*	Sum of squares	Variance components	% variation	Φ‐statistic (*p*‐value)
mtDNA cytochrome *b*					
Among populations	2	1,055.654	4.183 Va	39.63	Φ_CT_ **0.396** (.021 ± .01)
Among pop within groups	6	240.024	0.897 Vb	8.50	Φ_SC_ **0.141** (.000 ± .00)
Within populations	354	1937.945	5.474 Vc	51.87	Φ_ST_ **0.481** (.000 ± .00)
Total	362	3,233.623	10.554		
nDNA microsatellite loci					
Among populations	2	722.758	1.380 Va	23.58	Φ_CT_ **0.236** (.006 ± .00)
Among pop within groups	6	155.571	0.294 Vb	5.01	Φ_SC_ **0.066** (.000 ± .00)
Among individuals within populations	350	1507.118	0.126 Vc	2.15	Φ_IS_ **0.030** (.002 ± .00)
Within individuals	359	1,455.500	4.054 Vd	69.26	Φ_IT_ **0.307** (.000 ± .00)

Significant *p*‐values indicated in bold.

All AMOVA fixation indices were significant at *p* < .05 (Bonferroni corrected) for cytochrome *b* (Table 3). Variance within populations was greatest (Φ_ST_ = 0.481, 51.87%). Variation among populations (Φ_CT_ = 0.396) explained less than half of the variation observed (Table 3), and variance among populations within species groups was smallest (Φ_SC_ = 0.141, 8.5%), highlighting species‐specific signals. Neutrality tests of Tajima's D and Fu's F_S_ revealed Kimbe Bay *A. chrysopterus* and *A. sandaracinos* population size may be increasing or showing evidence of purifying selection at cytochrome *b*, indicated by significant (*p* < .05) negative Tajima's D (Table S4). Negative Fu's F_S_ for Kavieng hybrids suggests an excess number of haplotypes, as would be expected from recent population expansion; however, this was not found to be significant (where *p* < .02). All other populations displayed positive Fu's F_S_, suggesting a deficiency in haplotypes, as would be expected following a recent population bottleneck or over dominant selection (Fu, 1997). High haplotype diversity (h > 0.5) and low nucleotide diversity (π < 0.5) were recorded among parental and hybrid populations across the hybrid zone (Table 1), consistent with recent population expansions. Together these results provide evidence for a historical bottleneck followed by population expansion in the hybrid zone.

### 3.6 Contemporary genetic structure (nDNA)

Summary statistics for 21 microsatellite loci are presented in Table S3. Significant single‐locus departures from HWE were detected in 100 of 189 tests at population level before and 71 of 189 after sequential Bonferroni correction (a < 0.001; Table S3). Departure from HWE at locus As20, with homozygous excess revealed during analysis may be influenced by a null allele.

Genotypic diversity, based on microsatellite data, was moderate to high (0.478 ± 0.25 to 0.742 ± 0.37), with greater genotypic diversity estimates for hybrid populations compared with parent taxa across all three regions (Table 1). *Amphiprion chrysopterus* from Solomon Islands had the highest number of private alleles (P_a_ = 26), more than double that of Solomon Island hybrids and Kimbe Bay *A. sandaracinos* (11 and 10, respectively), which were next highest.

For all comparisons, population genetic differentiation was high (Tables 2, 3, and S5). Low estimates of actual differentiation (D_est_), between populations within species, indicate that region may not be important in structuring populations of parental and hybrid taxa (Table 2). There is a cascade of structure among taxa, where *A. sandaracinos* and *A. chrysopterus* were highly differentiated, *A. chrysopterus* and hybrids moderately differentiated and *A. sandaracinos* and hybrids least differentiated. This indicates species level is the most important factor structuring the various populations, despite ongoing hybridization and backcrossing (Table 2). Variation within individuals relative to the total was greatest (Φ_IT_ = 0.307, 69.25%), followed by variation among populations (Φ_CT_ = 0.236, 23.58%), based on AMOVA estimates (Table 3). Although significant, variation among populations within species groups and among individuals within populations contributed only 5.01% and 2.15%, respectively, to overall variation (Table 3).

Discriminant analysis of principle components visually defined clustering of populations in the hybrid zone (Figure 5b). *Amphiprion chrysopterus* populations grouped together and separated from all other populations along the x‐axis. Comparatively, *A. sandaracinos* and hybrid populations are differentiated along the y‐axis, where Kimbe Bay populations group loosely together and appear most different from the *A. chrysopterus* cluster. The Solomon Islands *A. sandaracinos* population was distinct from other populations, where Kavieng and Solomon Islands hybrid populations group together with Kavieng *A. sandaracinos* in the plot center (Figure 5b). Evidence of backcrossing and individual unique genotypes are indicated by dots falling outside 95% ellipses for all populations. The structure analysis used to inform DAPC supported two differentiated genetic clusters representing each parent species (Figures S5 and S6). When K = 2 clusters, an approximate 50% contribution of both parent species to hybrid populations is clearly defined in Kavieng and Solomon Islands regions (Figure 5c). In Kimbe Bay, closer to a 75% contribution to hybrids is evident from *A. sandaracinos*. Some individuals identified as *A. sandaracinos* were more similar to hybrids, providing evidence of ongoing backcrossing of hybrids with the smaller parent species in this region (Figure 5c). For comparison, when K = 3, a third cluster appears revealing Kimbe Bay hybrid and *A. sandaracinos* populations to be similar but differentiated from all other populations. This distinct cluster may result from ongoing backcrossing between these Kimbe Bay populations, where they are more genotypically similar to each other than to their conspecific populations (Figure 5c).

### 3.7 Genetic structure relative to parent species abundance

The degree to which parent species nDNA contributed to hybrid populations varied regionally regardless of the relative abundance of species. In Kimbe Bay, where the abundance of each pure parent species and hybrids were near equal (Figure 3), there is an asymmetric 25:75 contribution by *A. chrysopterus* and *A. sandaracinos* to hybrid populations (Figure 5c). In Kavieng and Solomon Islands, there is a near 50:50 input by *A. chrysopterus* and *A. sandaracinos* (Figure 5c), despite relatively high and low abundance, respectively, of *A. sandaracinos* compared to *A. chrysopterus* at these two locations (Figure 3).

## 4 DISCUSSION

Regional disparities in parent species frequency and inherent size disparities between hybridizing species drive variation in the genetic structure among taxa across the *A. leucokranos* hybrid zone. The relative abundance of parent species and hybrids varied across the hybrid zone regionally and observed levels of cohabitation did not reflect a scenario whereby rare species ‘seek out’ heterospecific mates in the absence of conspecifics. Subsequently, hybrid phenotypes were highly variable across the hybrid zone, reflecting the degree of backcrossing among hybrids and parent species relative to region. mtDNA revealed unidirectional hybridization among species, where the larger species was consistently female, and the smaller species was consistently male when interbreeding. Species level was most significant in structuring populations based on nDNA microsatellites, despite ongoing hybridization and persistent backcrossing throughout the hybrid zone, where two genetic clusters representing the parent species were defined. In contrast, the degree to which parent species nDNA contributed to hybrid populations varied regionally regardless of species relative abundances, with an asymmetric 25:75 contribution in Kimbe Bay, and 50:50 input elsewhere by *A. chrysopterus* and *A. sandaracinos*, respectively. This may reflect the extent of backcrossing in each region. High haplotypic diversity and low nucleotide diversity in all populations indicate that, historically, a bottleneck followed by a population expansion may have contributed to generation and subsequent expansion of the hybrid zone. Collectively, results suggest the hybrid (originally described as *A. leucokranos*) is no less fit than the parent species are and may persist in the hybrid zone to differentiate completely from parent species over time. This study shows that the outcome of hybridization is dependent on the social and ecological context in which taxa hybridize.

### 4.1 Regionally disparate abundance and cohabitation

In Kavieng and Solomon Island regions, where abundance disparities between parent species are evident, significantly more mixed species group assemblages occur than in Kimbe Bay, where conspecific groups are twice as common. In contrast, the frequency of each parent species in Kimbe Bay is relatively equal and overall conspecific assemblages predominate. Abundance disparities between species are considered a key factor facilitating hybridization between sister taxa in regions of range overlap (Hubbs, 1955). In a recent review of fish hybridization, rarity of one or both parent species was reported as the primary ecological factor implicated in promoting hybridization among marine fishes (Montanari et al., 2016). This was followed by shared resource use, specifically the degree of habitat and dietary overlap; however, these factors are not often empirically tested and rather proposed to explain this phenomenon. Mate choice experiments on hybridizing marine fishes are not currently available; however, experimentally altering the relative abundance of two largely sympatric grasshopper species increased hybridization propensity when relative frequencies of sister taxa were increasingly disparate, due to additional inter‐species encounters (Rohde, Hau, Weyer, & Hochkirch, 2015). Authors concluded that abundance disparities are a major driver of hybridization and experimentally found for the first time that hybridization probability increased with decreasing relative frequency of conspecific taxa (Rohde et al., 2015). Hybrid systems in which one species is rare and the other abundant are widely reported, where rare species are generally purported to choose mates from an abundant sister species in the absence of conspecifics (Allen, 1979; Frisch & van Herwerden, 2006; Hobbs & Allen, 2014; Marie et al., 2007; Montanari, Hobbs, Pratchett, Bay, & van Herwerden, 2014; Moyer, 1981; Randall, Allen, & Steene, 1977; van Herwerden et al., 2006). Within the *A. leucokranos* hybrid zone, the less abundant species did not consistently have a greater propensity for cohabitation with the more abundant species. For example, *A. chrysopterus* was more abundant than *A. sandaracinos* in Kavieng, and less abundant than *A. sandaracinos* in the Solomon Islands, yet showed a relatively greater propensity for cohabitation and hybridization with other taxa at both locations. This resulted regardless of who the most abundant species was, considering both mtDNA and nDNA exchange.

The data show that common species mate with less common species. Pyle and Randall (1994) asserted that the general assumption of rare species seeking out heterospecific mates does not consider why individuals from a common species might choose to mate with individuals from a rare species when conspecifics are abundant. It was suggested that particular social systems may provide alternative opportunities for reproduction at more favorable times for dominant individuals of a particular sex (Pyle & Randall, 1994), such as in the harem forming *Centropyge* species that hybridize (Kosaki, Pyle, Randall, & Irons, 1991; Lutnesky, 1992a, 1992b; Moyer, 1981, 1990). However, in *Centropyge* spp., gender frequency disparities are apparently more important drivers than species abundance disparities.

Here, we propose that in the *A. leucokranos* hybrid zone, where abundance disparities clearly appear to be associated with hybridization propensity, the underlying reason that abundant *A. chrysopterus* choose to hybridize may be associated with demand for a limited resource—the host anemone on which groups live and reproduce. In this hybrid zone, intraspecific competition for limited host anemones is great, and the larger species in a given scenario holds a significant size advantage when joining and living in mixed groups.

### 4.2 Drivers of population structure across this hybrid zone

What is driving the structure found across the *A. leucokranos* hybrid zone, where abundance disparities appear to promote hybridization? In considering preferences for conspecific or interspecific group formation, it is widely assumed that all individuals have equal choice in determining breeding partners. However, the assumption that mate choice is a level playing field in hybridization between species in hierarchical groups is fundamentally false, as the factor on which dominance depends may not be equally distributed among taxa (Bronson, Grubb, Sattler, & Braun, 2003; Reudink, Mech, & Curry, 2006). In the case of anemonefish, dominance is dependent on size, and anemonefish are well known for living in hierarchical groups in which size dominance determines an individual's right to reproduce as either a female or male (Buston, 2003, 2004; Buston & Cant, 2006; Fricke & Fricke, 1977; Moyer & Nakazono, 1978). In the case of hybridizing anemonefish, Gainsford et al. (2015) found that the maximum size of hybridizing taxa drives which species reproduces as the dominant female or subdominant male in mixed species group assemblages. Based on previous research across the *A. leucokranos* hybrid zone, it is likely that the bigger (i.e., dominant) fish always gets first choice of a mate. At all locations sampled, *A. chrysopterus* was always the larger species and apparently prefers conspecifics followed in choice by intermediately sized hybrids, and last—smaller *A. sandaracinos*. As in many group forming fish species, size of individuals can be particularly important in shaping ecological interactions. Reproductive success is highly skewed toward socially dominant individuals due to greater size, aggression, and fitness, thus attaining greater access to mates and limited resources (Ang & Manica, 2010; Keller & Reeve, 1994; Reeve & Keller, 2001; Vehrencamp, 1983; Wong, 2011). In this way, moderately sized hybrids and small *A. sandaracinos* are disadvantaged against the more dominant species and must continue to queue in the hope of reproducing, rather than facing eviction and becoming vulnerable to mortality outside the group (Buston, 2004; Wong, Munday, Buston, & Jones, 2008). Accordingly, these individuals may benefit from remaining in queues to maintain their reproductive position, through reduced eviction risk and shorter queue times, rather than recruiting to other groups (Mitchell, 2005; Wong, Buston, Munday, & Jones, 2007), and that may compensate for and potentially overcome size disadvantages in fish social hierarchies (Alcazar, Hilliard, Becker, Bernaba, & Fernald, 2014). However, it is conceivable that smaller nonbreeding *A. chrysopterus* in the wild may choose to “skip the queue” to breed, by relocating to nearby *A. sandaracinos* or hybrid inhabited anemones (if present), thereby gaining breeding rights—as one of the larger fish—and producing offspring (albeit hybrid). This would be an effective evolutionary strategy if hybrid fitness is comparable to purebred fitness. To test this hypothesis, future studies should quantify hybrid and parent species fitness in this system, as was done for butterflyfish hybrids and parent species at the Christmas island suture zone (Montanari et al., 2017) and experimentally test whether queue's function as expected in a hybrid context.

Smaller hybrid and *A. sandaracinos* mate preferences are not evident in the ecological data, as results retain the signature of larger species mate choice throughout the hybrid zone. We hypothesize that hybrids may prefer to mate with other hybrids, if available, but due to rarity would preferentially cohabit with the relatively smaller, subdominant species.

The influence of size dominance on gene flow is evident in genetic structure found among populations. Species level was most important in structuring populations; however, as size is not independent of taxonomic status in this hybridization scenario, it is proposed that the size of the parent species, rather than the species itself, is driving the direction of gene flow among species. mtDNA reflects a pattern of haplotype introgression from larger *A. chrysopterus* to *A. sandaracinos* via the intermediately sized hybrid conduit. When mixed species mated, hybrids appeared more genetically similar to the larger, dominant parent species. Based on maternally inherited DNA, this larger, dominant species would exclusively be the mother. Therefore, hybrid phenotypic diversity was ultimately influenced by the proportion of mixed species groups within each region, in addition to the taxonomic assemblage of groups. In this way, parent species size was important in shaping observed hybrid phenotypes due to the influence of anemonefish hierarchical behavior.

Genetic data also revealed two defined parental clusters, representing the larger *A. chrysopterus* and smaller *A. sandaracinos*, respectively. As would be expected when examining nuclear loci, hybrid populations have an intermediate 50:50 contribution of each parent species, except in Kimbe Bay where more of the hybrid genotypes are similar to the *A. sandaracinos* parent based on structure assignment. This apparently highlights extensive backcrossing among hybrid and *A. sandaracinos* populations in Kimbe Bay, where hybridization has most likely been occurring for longer than other regions sampled as suggested by the level of introgression. Moreover, this extensive backcrossing may also result from queue‐jumping behavior, thereby giving putative queue‐jumpers a fitness advantage, consistent with the observed level of backcrossing. This is not to say that backcrossing is not extensive in other regions. DAPC analysis showed, based on 21 highly polymorphic nDNA loci, that hybrid phenotypes are more *A. sandaracinos*‐like in each region, reflecting the hybrid choice (in the absence of other hybrids) to mate as the larger dominant female with the subdominant parent species males. An exception to this generalization is that in the Solomon Islands *A. sandaracinos* appears isolated from other taxa and is particularly rare. This in itself could result from queue‐jumping leading to the observed increase in hybrid and backcrossed anemonefishes (82% of total sampled in SO, compared to 35 and 40%, respectively in KB and KA) and may eventually lead to the disappearance of *A. sandaracinos* from the Solomon Islands.

### 4.3 Persistence of hybrid *A. leucokranos*

High haplotype and low nucleotide diversities throughout the hybrid zone suggest that hybridizing species have historically experienced a population bottleneck followed by rapid population growth, which has led to an accumulation of mutations (Avise, Neigel, & Arnold, 1984; Grant & Bowen, 1998; Rogers & Harpending, 1992). Grant and Bowen (1998) categorized such scenarios as examples of species which contain dominant haplotypes connected to clusters of unique haplotypes by only a few mutations and are mostly evolutionarily ‘young’ species. Recently diverged sister species reveal the association between biogeographical barriers and evolutionary patterns. For example, the evolutionary trajectories of many Indo‐Pacific marine fauna are directly related to glacial sea level fluctuations during the Pleistocene, which divided the Red Sea, Indian, and Pacific Oceans (DiBattista et al., 2016; McMillan & Palumbi, 1995; Palumbi, 1994; Timm & Kochzius, 2008). Anemonefish species studied in the Indo‐Pacific are highly related based on morphometrics, phylogenetic, and population genetic data (Gainsford et al., 2015; Timm et al., 2008). Specifically, *A. leucokranos* hybrids regardless of region have greater genotypic diversity than parent species. Hybrid zones, where the process of divergence is underway, offer insights into the importance of biogeography and ecology in shaping population histories and future evolutionary patterns. Despite drawing the conclusion that *A. leucokranos* may be a true species, Santini and Polacco (2006) concluded the probable area of origin for Amphiprionidae is the Coral Triangle, beginning somewhere between the Philippines and the Great Barrier Reef on east coast Australia, and Sumatra and Melanesia. This finding agrees with the Coral Triangle being the most significant hot spot for biodiversity and evolution of endemism (Roberts et al., 2002). Thus, it is not surprising that the hybrid zone examined here is located within the Coral Triangle, where Amphiprionidae first appeared and diversified (Litsios & Salamin, 2014), and where adaptive radiation of species through hybridization continues. Size‐based behavior, limiting bidirectional gene flow, and hybrid zone location along species distribution boundaries may contribute to *A. chrysopterus* and *A. sandaracinos* not merging, while promoting hybridization when abundance disparities exist.

The persistence of the hybrid *A. leucokranos* is associated with three key factors, which may contribute to speciation through time. Firstly, hybrid–hybrid pairs with egg clutches are consistently found, where offspring are viable based on both phenotypic and genetic evidence. Throughout the hybrid zone, hybrids regularly share resources with parent species, facilitating backcrossing with parent taxa, particularly with the smaller, subdominant *A. sandaracinos*. Back‐crosses in the other direction (with dominant *A. chrysopterus*) are also evident, albeit rare, due to the size‐dominant behavior structuring anemonefish groups.

There is a strong case for recognizing the status of this hybrid as more than an evolutionary dead end in light of overwhelming evidence for the importance of hybridization to the two parent taxa, as well as the indication that *A. leucokranos* appears to be differentiating from parent taxa. Recently, authors have highlighted the importance of acknowledging hybrid species in light of legislation which is inherently vague and generally does not consider protection or conservation policy measures (Allendorf, Leary, Spruell, & Wenburg, 2001; Chan, Hoffmann, & van Oppen, 2019; Richards & Hobbs, 2015). Losses of taxonomic evolutionary novelty and phylogenetic diversity, as well as increased species extinctions are predicted from inadequate management of hybridization and hybridizing lineages (Chunco, 2014; Dowling & Secor, 1997; Forest et al., 2007; Van Dyke, 2008). Pertinently, *A. leucokranos*, a highly prized aquarium trade species that is iconic, rare and easily caught due to reliance on sessile anemone hosts, is likely to be detrimentally impacted by removal of its current species status. Taxonomic delisting of this species, already prized by aquarium traders, may lead to increased harvest and rarity of this already locally rare and endemic taxon, simultaneously driving an increase in market value of individual fish and hence greater motivation for trade. Richards and Hobbs (2015) concluded that in order to conserve coral reef biodiversity, and the processes that are implicit in initiating and maintaining biodiversity, such as hybridization, policies regarding conservation and management must be addressed on an individual case basis, as removal of species status or lack of protection may indirectly impact evolution and biodiversity of species overall.

Extensive investigation of the *Amphiprion leucokranos* hybrid zone revealed that parent species abundance and size disparities drive regional ecological patterns and genetic structure among taxa. The size of parent species, rather than the species itself, better explains the historical and existing genetic structure, reflecting the characteristic size‐based dominance behavior of anemonefish. This study demonstrated that rare species may not always choose to hybridize with abundant species when abundance disparities arise, such as along the edges of their biogeographical distributions. High haplotypic diversity and low nucleotide diversity in all populations examined suggest a bottleneck followed by recent population expansion that has led to initiation and persistence of this hybrid zone, where the hybrid *A*. *leucokranos* appears to be differentiating from the parent taxa. This study emphasizes the need and importance of protection for hybrid species. Not only are *A. leucokranos* vulnerable to over‐harvesting by aquarium traders, but they are also important contributors to both the evolutionary resilience of hybridizing parent species and the biodiversity of coral reef systems.

## CONFLICT OF INTEREST

The authors of this manuscript declare no conflict of interest, financial, or otherwise.

## AUTHOR CONTRIBUTIONS

A.G., L.V.H., and G.P.J.: Study conception and design. G.P.J., A.G., L.V.H., and J.P.H.: Funding and resources. A.G., F.M.H., and L.V.H.: Research. A.G., L.V.H., and G.P.J.: Data analysis. A.G.: Original manuscript writing and all authors: Revisions.

## Supporting information

Supplementary Material

## Data Availability

Cytochrome *b* sequences available from NCBI GenBank: accession numbers MN150716—MN151076. Microsatellite genotype data available from the Dryad Digital Repository: https://doi.org/10.5061/dryad.f5hf2fn.
