# Supplementary material for: Species integrity, introgression, and genetic variation across a coral reef fish hybrid zone
Source: Ecol Evol. 2020 Oct 21;10(21):11998–2014. doi: 10.1002/ece3.6769 (PMC7663085; doi:10.1002/ece3.6769)
Supplement: Supplementary file 1 — Supplementary Material [file ECE3-10-11998-s001.docx]

**Supplementary Tables & Figures**

| Table S1 Phenotypic expectations for taxa across hybrid zone  Table S2 Qualitative phenotypic traits observed in hybrid zone  Table S3 Summary statistics for 21 microsatellite loci across all populations  Table S4 mtDNA neutrality tests: Tajima’s D and Fu’s FS  Table S5 Raw species differentiation from microsatellite allele frequencies  Figure S1 *Amphiprion chrysopterus* hybrid zone phenotypes |
| --- |

**Table S1** Phenotype expectations for pure *A. chrysopterus* (CH), *A. chrysopterus* back-crosses (CH b/c), first generation hybrids (F1 hybrid), *A. sandaracinos* back-crosses (SA b/c), and pure *A. sandaracinos* (SA), throughout the hybrid zone. Pigmentation within the Solomon Islands region is also expected for *A. chrysopterus* individuals, additional to this table.

| *Trait* | Pure CH | CH b/c | F1 hybrid | SA b/c | Pure SA |
| --- | --- | --- | --- | --- | --- |
| Tail shape | Elongated | Elongated | Round | Round | Round |
| Tail colour | White | White | Orange | Orange | Orange |
| Body colour | Black | Black/brown | Orange | Orange | Orange |
| Dorsal stripe | Absent | Absent | White cap | Partial white cap and / or stripe | Complete white stripe |
| 1^st^ side bars | Complete | Complete | Partial | Partial | Absent |
| 2^nd^ side bars | Complete | Partial/ complete | Absent | Absent | Absent |
| Lateral body shape | Deep | Deep | Deep | Narrow | Narrow |

**Table S2** Relative frequency of qualitative phenotypic traits observed in hybrid zone; where in CHKB and CHSO populations, 2% and 19%, respectively were light variants (see Figure S1C), and 9% showed pigmentation (see Figure S1A).

|  | CHKB | CHKA | CHSO | LUKB | LUKA | LUSO | SAKB | SAKA | SASO |
| --- | --- | --- | --- | --- | --- | --- | --- | --- | --- |
| Total *n* | 54 | 29 | 90 | 26 | 23 | 52 | 62 | 29 | 35 |
| Tail Shape   - 1. Round   2. Elongated | 0.00  1.00 | 0.00  1.00 | 0.00  1.00 | 0.96  0.04 | 0.96  0.04 | 0.04  0.96 | 1.00  0.00 | 1.00  0.00 | 1.00  0.00 |
| Tail Colour   1. White 2. Orange 3. Brown 4. Black | 1.00  0.00  0.00  0.00 | 1.00  0.00  0.00  0.00 | 0.95  0.00  0.02  0.03 | 0.04  0.96  0.00  0.00 | 0.09  0.91  0.00  0.00 | 0.08  0.92  0.00  0.00 | 0.00  1.00  0.00  0.00 | 0.00  1.00  0.00  0.00 | 0.00  1.00  0.00  0.00 |
| Body Colour   1. Black 2. Brown 3. Orange | 0.94  0.06  0.00 | 0.52  0.48  0.00 | 0.79  0.21  0.00 | 0.00  0.12  0.88 | 0.00  0.13  0.87 | 0.00  0.17  0.83 | 0.00  0.00  1.00 | 0.00  0.00  1.00 | 0.00  0.00  1.00 |
| Dorsal Stripe   1. Cap 2. Full 3. Partial 4. Missing | 0.00  0.00  0.00  1.00 | 0.00  0.00  0.00  1.00 | 0.00  0.00  0.00  1.00 | 0.42  0.12  0.04  0.42 | 0.70  0.04  0.22  0.04 | 0.58  0.08  0.15  0.19 | 0.00  1.00  0.00  0.00 | 0.00  1.00  0.00  0.00 | 0.00  1.00  0.00  0.00 |
| 1^st^ Side Bars   1. Complete pair 2. Partial pair 3. Complete single 4. Partial single 5. Both missing | 0.98  0.02  0.00  0.00  0.00 | 1.00  0.00  0.00  0.00  0.00 | 1.00  0.00  0.00  0.00  0.00 | 0.31  0.54  0.00  0.04  0.12 | 0.65  0.30  0.00  0.00  0.04 | 0.58  0.38  0.00  0.04  0.00 | 0.00  0.02  0.00  0.00  0.98 | 0.00  0.00  0.00  0.00  1.00 | 0.00  0.00  0.00  0.00  1.00 |
| 2^nd^ Side Bars   1. Complete pair 2. Partial pair 3. Complete single 4. Partial single 5. Both missing | 0.98  0.02  0.00  0.00  0.00 | 0.90  0.10  0.00  0.00  0.00 | 0.94  0.06  0.00  0.00  0.00 | 0.00  0.00  0.00  0.00  1.00 | 0.00  0.04  0.00  0.00  0.96 | 0.02  0.00  0.00  0.02  0.96 | 0.00  0.00  0.00  0.00  1.00 | 0.00  0.00  0.00  0.00  1.00 | 0.00  0.00  0.00  0.00  1.00 |
| Lateral Body Shape   1. Narrow 2. Deep | 0.00  1.00 | 0.00  1.00 | 0.00  1.00 | 0.42  0.58 | 0.13  0.87 | 0.37  0.63 | 1.00  0.00 | 0.93  0.07 | 1.00  0.00 |

**Table S3** Summary statistics for 21 microsatellite loci across nine populations: Sample size (n), observed number of alleles (N_a_) and private alleles (P_a_), observed heterozygosity (H_O_) expected heterozygosity (H_E_), and average inbreeding coefficient (*F*_IS_). Probability of departure from HWE for each locus at each species (*P*); where significance of departure before *P*< 0.05* and following sequential Bonferroni adjustment *P <* 0.001 (bold) are indicated. Loci listed are as follows: Alat10, Alat23, D1, As20, Alat14, Alat16, D114, Alat11, Alat12, A130, Alat5, Alat21, Alat13, Alat7, Am9, Alat19, Alat8, Am21, Alat22, Am5, and As8.

|  |  | **1** | **2** | **3** | **4** | **5** | **6** | **7** | **8** | **9** | **10** | **11** | **12** | **13** | **14** | **15** | **16** | **17** | **18** | **19** | **20** | **21** |
| --- | --- | --- | --- | --- | --- | --- | --- | --- | --- | --- | --- | --- | --- | --- | --- | --- | --- | --- | --- | --- | --- | --- |
| CHKB | n | 30 | 30 | 29 | 31 | 30 | 31 | 31 | 22 | 31 | 29 | 31 | 31 | 29 | 31 | 30 | 28 | 29 | 29 | 31 | 30 | 30 |
|  | N_a_ | 7 | 4 | 15 | 3 | 2 | 13 | 17 | 11 | 7 | 15 | 6 | 9 | 6 | 12 | 17 | 16 | 12 | 8 | 5 | 7 | 6 |
|  | P_a_ | 0 | 0 | 0 | 1 | 0 | 0 | 0 | 1 | 2 | 1 | 1 | 1 | 0 | 3 | 0 | 0 | 1 | 0 | 0 | 0 | 1 |
|  | H_O_ | 0.567 | 0.133 | 0.586 | 0.032 | 0.000 | 0.839 | 0.871 | 0.364 | 0.581 | 0.897 | 0.613 | 0.194 | 0.552 | 0.677 | 0.900 | 0.857 | 0.862 | 0.586 | 0.774 | 0.500 | 0.300 |
|  | H_E_ | 0.658 | 0.243 | 0.908 | 0.122 | 0.064 | 0.888 | 0.906 | 0.849 | 0.589 | 0.857 | 0.669 | 0.745 | 0.680 | 0.682 | 0.873 | 0.907 | 0.837 | 0.807 | 0.535 | 0.636 | 0.472 |
|  | *F*_IS_ | 0.155 | 0.464 | 0.370 | 0.744 | 1.000 | 0.072 | 0.055 | 0.587 | 0.031 | -0.029 | 0.100 | 0.747 | 0.205 | 0.023 | -0.014 | 0.073 | -0.012 | 0.290 | -0.433 | 0.230 | 0.379 |
|  | *P* | 0.029* | **0.000*** | **0.000*** | **0.000*** | **0.000*** | 0.363 | 0.209 | **0.000*** | **0.000*** | 0.063 | **0.000*** | **0.000*** | **0.000*** | 0.762 | **0.000*** | 0.016* | 0.572 | 0.132 | **0.000*** | 0.005* | **0.000*** |
| CHKA | n | 26 | 26 | 26 | 26 | 26 | 26 | 26 | 21 | 26 | 26 | 26 | 26 | 24 | 26 | 26 | 24 | 26 | 26 | 26 | 26 | 25 |
|  | N_a_ | 5 | 3 | 13 | 1 | 1 | 14 | 25 | 9 | 3 | 11 | 7 | 7 | 4 | 8 | 17 | 14 | 10 | 11 | 2 | 7 | 7 |
|  | P_a_ | 0 | 0 | 0 | 0 | 0 | 0 | 4 | 2 | 0 | 0 | 1 | 2 | 0 | 0 | 0 | 0 | 1 | 0 | 0 | 0 | 0 |
|  | H_O_ | 0.577 | 0.231 | 0.654 | 0.000 | 0.000 | 0.923 | 1.000 | 0.381 | 0.538 | 0.923 | 0.923 | 0.346 | 0.583 | 0.692 | 0.846 | 0.917 | 0.885 | 0.769 | 0.885 | 0.423 | 0.360 |
|  | H_E_ | 0.490 | 0.211 | 0.891 | 0.000 | 0.000 | 0.865 | 0.940 | 0.783 | 0.533 | 0.851 | 0.667 | 0.753 | 0.608 | 0.749 | 0.902 | 0.890 | 0.848 | 0.839 | 0.493 | 0.547 | 0.466 |
|  | *F*_IS_ | -0.157 | -0.075 | 0.284 | - | - | -0.048 | -0.044 | 0.531 | 0.010 | -0.065 | -0.367 | 0.554 | 0.061 | 0.095 | 0.081 | -0.009 | -0.024 | 0.102 | -0.786 | 0.246 | 0.246 |
|  | *P* | 0.982 | 0.931 | **0.000*** | - | - | 0.151 | 0.600 | **0.000*** | 0.996 | 0.431 | 0.456 | **0.000*** | 0.525 | 0.653 | 0.171 | 0.314 | 0.490 | 0.402 | **0.000*** | 0.101 | 0.006* |
| CHSO | n | 65 | 63 | 62 | 61 | 63 | 64 | 52 | 58 | 64 | 63 | 65 | 65 | 55 | 61 | 63 | 60 | 61 | 56 | 65 | 63 | 64 |
|  | N_a_ | 12 | 7 | 17 | 1 | 2 | 17 | 35 | 14 | 5 | 16 | 10 | 11 | 9 | 12 | 24 | 19 | 16 | 10 | 8 | 13 | 8 |
|  | P_a_ | 3 | 2 | 1 | 0 | 0 | 1 | 5 | 2 | 0 | 1 | 2 | 3 | 1 | 2 | 6 | 0 | 3 | 0 | 3 | 0 | 1 |
|  | H_O_ | 0.708 | 0.270 | 0.613 | 0.000 | 0.000 | 0.938 | 0.923 | 0.207 | 0.438 | 0.905 | 0.785 | 0.338 | 0.709 | 0.623 | 0.889 | 0.917 | 0.852 | 0.732 | 0.708 | 0.762 | 0.359 |
|  | H_E_ | 0.754 | 0.322 | 0.902 | 0.000 | 0.031 | 0.875 | 0.947 | 0.845 | 0.559 | 0.844 | 0.703 | 0.790 | 0.714 | 0.620 | 0.913 | 0.924 | 0.866 | 0.816 | 0.497 | 0.722 | 0.576 |
|  | *F*_IS_ | 0.069 | 0.171 | 0.328 | - | 1.000 | -0.064 | 0.035 | 0.759 | 0.225 | -0.064 | -0.109 | 0.577 | 0.016 | 0.004 | 0.035 | 0.017 | 0.024 | 0.112 | -0.416 | -0.047 | 0.383 |
|  | *P* | **0.000*** | **0.000*** | 0.002* | - | **0.000*** | 0.047* | 0.303 | **0.000*** | **0.000*** | 0.517 | **0.000*** | **0.000*** | **0.000*** | 0.005* | 0.123 | **0.000*** | 0.007* | **0.000*** | **0.000*** | **0.000*** | **0.000*** |
| LUKB | n | 34 | 35 | 34 | 34 | 35 | 35 | 35 | 34 | 35 | 34 | 35 | 35 | 35 | 33 | 35 | 33 | 35 | 34 | 35 | 35 | 35 |
|  | N_a_ | 6 | 4 | 13 | 4 | 2 | 14 | 11 | 5 | 3 | 7 | 6 | 7 | 3 | 6 | 12 | 15 | 9 | 9 | 9 | 7 | 3 |
|  | P_a_ | 0 | 0 | 0 | 0 | 0 | 1 | 0 | 0 | 0 | 0 | 0 | 0 | 0 | 0 | 0 | 0 | 0 | 0 | 0 | 0 | 0 |
|  | H_O_ | 0.971 | 0.571 | 0.824 | 0.559 | 0.486 | 1.000 | 0.829 | 0.088 | 0.629 | 0.647 | 0.657 | 0.514 | 0.114 | 0.455 | 0.943 | 0.758 | 0.686 | 0.794 | 0.914 | 0.657 | 0.257 |
|  | H_E_ | 0.771 | 0.446 | 0.863 | 0.431 | 0.396 | 0.818 | 0.871 | 0.333 | 0.589 | 0.667 | 0.661 | 0.760 | 0.160 | 0.385 | 0.749 | 0.862 | 0.706 | 0.723 | 0.790 | 0.580 | 0.564 |
|  | *F*_IS_ | -0.245 | -0.269 | 0.061 | -0.282 | -0.214 | -0.209 | 0.063 | 0.742 | -0.053 | 0.045 | 0.020 | 0.336 | 0.299 | -0.166 | -0.246 | 0.136 | 0.043 | -0.084 | -0.143 | -0.119 | 0.554 |
|  | *P* | **0.000*** | 0.469 | 0.314 | **0.000*** | 0.177 | 0.080 | 0.077 | **0.000*** | 0.094 | 0.999 | 0.027* | 0.001* | **0.000*** | 1.000 | 0.948 | 0.740 | 0.912 | 0.997 | 0.656 | 0.123 | **0.000*** |
| LUKA | n | 25 | 25 | 25 | 25 | 25 | 25 | 25 | 24 | 24 | 25 | 25 | 25 | 25 | 25 | 25 | 25 | 25 | 24 | 25 | 25 | 25 |
|  | N_a_ | 7 | 4 | 19 | 2 | 2 | 16 | 19 | 6 | 4 | 14 | 5 | 9 | 4 | 9 | 14 | 16 | 11 | 8 | 12 | 9 | 4 |
|  | P_a_ | 0 | 0 | 0 | 0 | 0 | 0 | 1 | 1 | 0 | 0 | 0 | 0 | 0 | 1 | 0 | 0 | 0 | 0 | 0 | 0 | 0 |
|  | H_O_ | 0.800 | 0.840 | 0.880 | 0.920 | 0.840 | 1.000 | 0.880 | 0.167 | 0.833 | 1.000 | 0.800 | 0.320 | 0.160 | 0.880 | 0.960 | 1.000 | 0.880 | 0.833 | 0.880 | 0.880 | 0.320 |
|  | H_E_ | 0.714 | 0.551 | 0.911 | 0.497 | 0.500 | 0.888 | 0.910 | 0.661 | 0.672 | 0.839 | 0.656 | 0.846 | 0.545 | 0.722 | 0.863 | 0.924 | 0.866 | 0.828 | 0.665 | 0.758 | 0.618 |
|  | *F*_IS_ | -0.100 | -0.509 | 0.055 | -0.846 | -0.669 | -0.106 | 0.053 | 0.757 | -0.220 | -0.172 | -0.200 | 0.634 | 0.716 | -0.199 | -0.092 | -0.062 | 0.004 | 0.015 | -0.305 | -0.142 | 0.498 |
|  | *P* | **0.000*** | 0.053 | 0.012* | **0.000*** | 0.001* | 0.887 | 0.978 | **0.000*** | 0.608 | 0.074 | 0.111 | **0.000*** | **0.000*** | 0.681 | 0.913 | 0.100 | 0.385 | 0.564 | 1.000 | 0.594 | **0.000*** |

**Table S3** Continued.

|  |  | **1** | **2** | **3** | **4** | **5** | **6** | **7** | **8** | **9** | **10** | **11** | **12** | **13** | **14** | **15** | **16** | **17** | **18** | **19** | **20** | **21** |
| --- | --- | --- | --- | --- | --- | --- | --- | --- | --- | --- | --- | --- | --- | --- | --- | --- | --- | --- | --- | --- | --- | --- |
| LUSO | n | 52 | 53 | 47 | 53 | 53 | 53 | 52 | 48 | 53 | 53 | 53 | 53 | 53 | 53 | 53 | 52 | 53 | 22 | 23 | 53 | 53 |
|  | N_a_ | 10 | 6 | 22 | 5 | 2 | 17 | 24 | 9 | 6 | 21 | 10 | 15 | 7 | 11 | 18 | 25 | 18 | 9 | 11 | 8 | 4 |
|  | P_a_ | 0 | 0 | 0 | 0 | 0 | 0 | 1 | 1 | 0 | 1 | 3 | 3 | 2 | 1 | 0 | 0 | 3 | 0 | 1 | 0 | 0 |
|  | H_O_ | 0.269 | 0.887 | 0.851 | 0.849 | 0.698 | 0.943 | 0.731 | 0.229 | 0.585 | 0.981 | 0.755 | 0.415 | 0.113 | 0.811 | 0.962 | 1.000 | 0.962 | 0.818 | 0.826 | 0.962 | 0.075 |
|  | H_E_ | 0.727 | 0.602 | 0.941 | 0.530 | 0.497 | 0.896 | 0.878 | 0.810 | 0.593 | 0.912 | 0.686 | 0.848 | 0.718 | 0.653 | 0.818 | 0.943 | 0.867 | 0.830 | 0.680 | 0.766 | 0.537 |
|  | *F*_IS_ | 0.636 | -0.465 | 0.106 | -0.596 | -0.396 | -0.044 | 0.177 | 0.722 | 0.024 | -0.066 | -0.090 | 0.517 | 0.845 | -0.234 | -0.167 | -0.051 | -0.100 | 0.037 | -0.194 | -0.248 | 0.862 |
|  | *P* | **0.000*** | 0.003* | 0.001* | **0.000*** | 0.003* | 0.830 | 0.943 | **0.000*** | 0.049* | 0.301 | **0.000*** | **0.000*** | **0.000*** | 0.989 | 0.011* | 0.042* | 0.094 | 0.126 | 0.370 | **0.000*** | **0.000*** |
| SAKB | n | 65 | 64 | 49 | 60 | 62 | 63 | 60 | 56 | 66 | 62 | 65 | 64 | 64 | 65 | 65 | 59 | 63 | 60 | 66 | 66 | 65 |
|  | N_a_ | 7 | 4 | 11 | 2 | 3 | 12 | 13 | 8 | 7 | 6 | 5 | 9 | 4 | 6 | 8 | 13 | 6 | 7 | 16 | 8 | 5 |
|  | P_a_ | 1 | 0 | 1 | 0 | 1 | 0 | 0 | 0 | 1 | 1 | 1 | 1 | 0 | 0 | 0 | 2 | 0 | 0 | 1 | 0 | 0 |
|  | H_O_ | 0.815 | 0.063 | 0.735 | 0.050 | 0.048 | 0.810 | 0.933 | 0.161 | 0.515 | 0.532 | 0.292 | 0.656 | 0.047 | 0.185 | 0.585 | 0.797 | 0.508 | 0.400 | 0.712 | 0.212 | 0.600 |
|  | H_E_ | 0.735 | 0.061 | 0.847 | 0.049 | 0.047 | 0.738 | 0.846 | 0.359 | 0.577 | 0.515 | 0.370 | 0.734 | 0.159 | 0.213 | 0.632 | 0.842 | 0.566 | 0.564 | 0.808 | 0.273 | 0.543 |
|  | *F*_IS_ | -0.101 | -0.014 | 0.142 | -0.017 | -0.011 | -0.088 | -0.095 | 0.559 | 0.115 | -0.025 | 0.218 | 0.114 | 0.710 | 0.143 | 0.082 | 0.063 | 0.111 | 0.298 | 0.126 | 0.231 | -0.098 |
|  | *P* | 0.029* | 1.000 | **0.000*** | 0.843 | 0.998 | **0.000*** | 0.954 | **0.000*** | **0.000*** | **0.000*** | **0.000*** | **0.000*** | **0.000*** | **0.000*** | 0.175 | **0.000*** | 0.270 | 0.001* | **0.000*** | **0.000*** | **0.000*** |
| SAKA | n | 28 | 28 | 26 | 27 | 28 | 28 | 28 | 28 | 27 | 28 | 28 | 28 | 28 | 28 | 28 | 28 | 28 | 28 | 28 | 28 | 28 |
|  | N_a_ | 7 | 3 | 11 | 2 | 3 | 10 | 12 | 7 | 3 | 11 | 4 | 7 | 5 | 3 | 8 | 11 | 9 | 5 | 19 | 6 | 5 |
|  | P_a_ | 0 | 0 | 0 | 0 | 1 | 0 | 0 | 0 | 0 | 0 | 0 | 0 | 0 | 0 | 0 | 0 | 0 | 0 | 0 | 1 | 0 |
|  | H_O_ | 0.536 | 0.071 | 0.885 | 0.000 | 0.036 | 0.893 | 0.893 | 0.536 | 0.556 | 0.786 | 0.750 | 0.786 | 0.571 | 0.143 | 0.643 | 0.893 | 0.750 | 0.571 | 0.964 | 0.500 | 0.679 |
|  | H_E_ | 0.554 | 0.135 | 0.825 | 0.071 | 0.103 | 0.786 | 0.827 | 0.589 | 0.516 | 0.769 | 0.531 | 0.781 | 0.570 | 0.135 | 0.638 | 0.874 | 0.705 | 0.615 | 0.893 | 0.528 | 0.595 |
|  | *F*_IS_ | 0.052 | 0.486 | -0.052 | 1.000 | 0.663 | -0.118 | -0.062 | 0.108 | -0.057 | -0.003 | -0.397 | 0.012 | 0.015 | -0.043 | 0.011 | -0.003 | -0.045 | 0.089 | -0.061 | 0.071 | -0.123 |
|  | *P* | **0.000*** | **0.000*** | 0.324 | **0.000*** | **0.000*** | 0.002* | 0.002* | 0.047* | 0.717 | 0.006* | 0.121 | 0.001* | 0.920 | 0.983 | **0.000*** | **0.000*** | 0.111 | **0.000*** | **0.000*** | **0.000*** | 0.650 |
| SASO | n | 30 | 30 | 27 | 30 | 30 | 30 | 30 | 30 | 30 | 30 | 30 | 30 | 30 | 30 | 30 | 30 | 30 | 28 | 30 | 30 | 30 |
|  | N_a_ | 7 | 2 | 10 | 3 | 1 | 9 | 8 | 5 | 2 | 11 | 2 | 8 | 5 | 2 | 4 | 11 | 7 | 6 | 12 | 4 | 3 |
|  | P_a_ | 0 | 0 | 0 | 0 | 0 | 0 | 0 | 0 | 0 | 1 | 0 | 0 | 0 | 0 | 0 | 0 | 0 | 0 | 2 | 0 | 0 |
|  | H_O_ | 0.900 | 0.033 | 0.815 | 0.033 | 0.000 | 0.767 | 0.700 | 0.333 | 0.300 | 0.700 | 0.033 | 0.800 | 0.600 | 0.067 | 0.567 | 0.867 | 0.667 | 0.536 | 0.967 | 0.467 | 0.400 |
|  | H_E_ | 0.704 | 0.033 | 0.834 | 0.156 | 0.000 | 0.741 | 0.679 | 0.474 | 0.473 | 0.753 | 0.033 | 0.740 | 0.624 | 0.064 | 0.552 | 0.885 | 0.669 | 0.621 | 0.849 | 0.534 | 0.504 |
|  | *F*_IS_ | -0.262 | 0.000 | 0.042 | 0.793 | - | -0.018 | -0.013 | 0.313 | 0.380 | 0.088 | 0.000 | -0.064 | 0.056 | -0.018 | -0.010 | 0.038 | 0.020 | 0.154 | -0.121 | 0.143 | 0.223 |
|  | *P* | 0.844 | 0.926 | 0.910 | **0.000*** | - | 0.960 | 0.657 | 0.809 | 0.045* | 0.997 | 0.926 | 0.102 | 0.777 | 0.850 | 0.684 | 0.024* | 0.027* | 0.207* | 0.895 | 0.695 | 0.181 |

**Table S4** Neutrality tests using 1000 simulations of the infinite site model in Arlequin on mtDNA sequences from nine hybrid zone populations. Significant values in bold.

|  | CHKB | CHKA | CHSO | LUKB | LUKA | LUSO | SAKB | SAKA | SASO | Mean | s.d. |
| --- | --- | --- | --- | --- | --- | --- | --- | --- | --- | --- | --- |
| *Tajima’s D test* | | | | | | | | | | | |
| Sample size | 76 | 31 | 56 | 45 | 23 | 55 | 30 | 23 | 24 | 40.333 | 18.762 |
| S | 33 | 3 | 4 | 25 | 2 | 6 | 35 | 27 | 74 | 23.222 | 23.333 |
| Pi | 6.022 | 3.708 | 3.855 | 10.620 | 0.933 | 2.981 | 23.720 | 27.316 | 50.583 | 14.415 | 16.525 |
| Tajima's D | **-1.533** | -0.155 | -0.669 | -1.102 | 0.243 | -1.185 | **-1.875** | -0.554 | 1.593 | -0.582 | 1.049 |
| Tajima's D p-value | **0.038** | 0.468 | 0.290 | 0.128 | 0.698 | 0.100 | **0.012** | 0.300 | 0.966 | 0.333 | 0.324 |
| *Fu’s FS test* | | | | | | | | | | | |
| Real no. of alleles | 16 | 7 | 9 | 13 | 5 | 6 | 14 | 12 | 15 | 10.778 | 4.116 |
| Orig. no of alleles | 17 | 7 | 9 | 13 | 5 | 6 | 14 | 12 | 15 | 10.889 | 4.285 |
| Theta pi | 6.023 | 3.708 | 3.855 | 10.620 | 0.933 | 2.981 | 23.720 | 27.316 | 50.583 | 14.415 | 16.525 |
| Exp. no. of alleles | 16.205 | 8.761 | 11.062 | 17.997 | 3.589 | 9.348 | 19.672 | 16.917 | 19.803 | 13.706 | 5.707 |
| FS | 0.315 | 1.716 | 1.625 | 3.486 | -1.117 | 2.969 | 5.020 | 5.292 | 5.658 | 2.774 | 2.342 |
| Significance at p<0.02 | ns | ns | ns | ns | ns | ns | ns | ns | ns | ns | ns |

**Table S5** Raw species differentiation from microsatellite allele frequencies**:** species differentiation corrected for null allele frequencies using the ENA correction, where values significant to the 95% confidence interval are in bold; and estimator of actual differentiation (D_est_). Results are presented locus-by-locus and as an average over 21 loci and reveal comparable raw values to values corrected for null alleles.

| Locus | Raw | ENA corrected | D_est_ |
| --- | --- | --- | --- |
| Alat10_tri | 0.164 | 0.163 | 0.482 |
| Alat23_penta | **0.523** | **0.498** | 0.442 |
| D1_tetra | **0.061** | **0.058** | 0.565 |
| As20_tri | **0.644** | **0.610** | 0.435 |
| Alat14_tri | **0.675** | **0.630** | 0.440 |
| Alat16_tetra | **0.103** | **0.103** | 0.597 |
| D114_tetra | **0.050** | **0.051** | 0.422 |
| Alat11_tri | 0.207 | 0.186 | 0.476 |
| Alat12_tri | **0.119** | **0.111** | 0.173 |
| A130_di | **0.135** | **0.132** | 0.509 |
| Alat5_di | 0.232 | 0.225 | 0.393 |
| Alat21_penta | **0.114** | **0.100** | 0.493 |
| Alat13_tri | 0.200 | 0.178 | 0.249 |
| Alat7_di | **0.315** | **0.307** | 0.402 |
| Am9_tri | **0.116** | **0.114** | 0.459 |
| Alat19_tetra | **0.049** | **0.048** | 0.501 |
| Alat8_di | **0.128** | **0.124** | 0.484 |
| Am21_penta | **0.130** | **0.117** | 0.402 |
| Alat22_penta | 0.195 | 0.195 | 0.504 |
| Am5_di | **0.313** | **0.295** | 0.632 |
| As8_tetra | 0.271 | 0.239 | 0.460 |
| Mean | 0.204 | 0.194 | 0.450 |


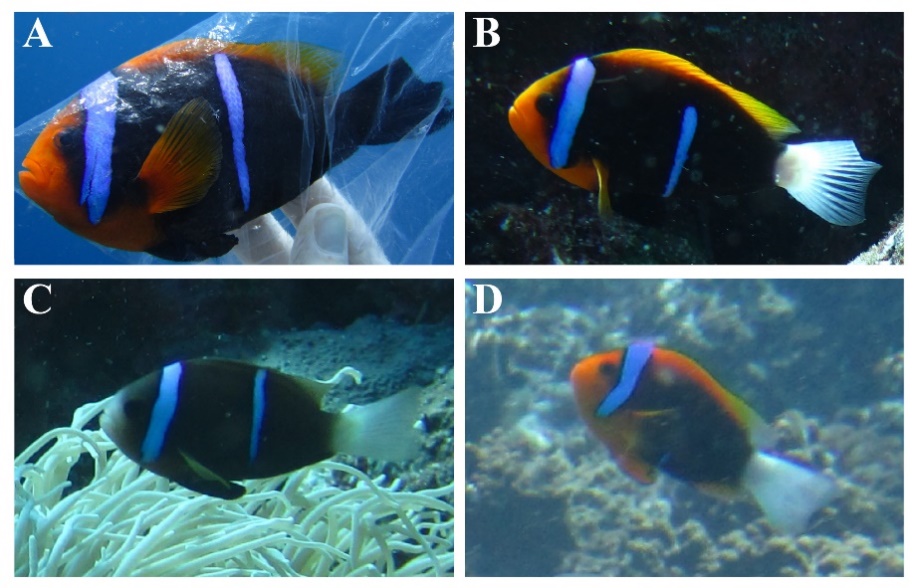


**Figure S1** *A. chrysopterus* hybrid zone phenotypes including: (A) black pigmented morph, (B) half second sidebar, and (C) light morph, New Georgia Province, Solomon Islands, and (D) putative *A. chrysopterus* & ‘*A. leucokranos*’ hybrid morph, Kimbe Bay, Papua New Guinea. Segments of caudal fin missing due to fin-clip sampling (A, B). Photo credits: A. Gainsford.
